# Coordination compounds containing bis-di­thiol­ene-chelated molybdenum(IV) and oxalate: comparison of terminal with bridging oxalate

**DOI:** 10.1107/S205698901701026X

**Published:** 2017-07-18

**Authors:** Agata Gapinska, Alan J. Lough, Ulrich Fekl

**Affiliations:** a Department of Chemical and Physical Sciences, University of Toronto, Mississauga, Ontario, L5L 1C6, Canada; b Department of Chemistry, 80 St George St., Toronto, ON, M5S 3H6, Canada

**Keywords:** crystal structure, di­thiol­ene, trigonal prismatic, oxalate

## Abstract

Two coordination compounds containing tetra-*n*-butyl­ammonium cations and bis-tfd-chelated molybdenum(IV) [tfd^2−^ = S_2_C_2_(CF_3_)_2_^2−^] and oxalate (ox^2−^, C_2_O_4_^2−^) in complex anions are reported, namely bis­(tetra-*n*-butyl­ammonium) bis­(1,1,1,4,4,4-hexa­fluoro­but-2-ene-2,3-di­thiol­ato)oxalatomolybdate(IV)–chloro­form–oxalic acid (1/1/1), (C_16_H_36_N)_2_[Mo(C_4_F_6_S_2_)_2_(C_2_O_4_)]·CHCl_3_·C_2_H_2_O_4_ or (N^*n*^Bu_4_)_2_[Mo(tfd)_2_(ox)]·CHCl_3_·C_2_H_2_O_4_, and bis­(tetra-*n*-butyl­ammonium) μ-oxalato-bis­[bis­(1,1,1,4,4,4-hexa­fluoro­but-2-ene-2,3-di­thiol­ato)molybdate(IV)], (C_16_H_36_N)_2_[Mo_2_(C_4_F_6_S_2_)_4_(C_2_O_4_)] or (N^*n*^Bu_4_)_2_[(tfd)_2_Mo(μ-ox)Mo(tfd)_2_]. They contain a terminal oxalate ligand in the first compound and a bridging oxalate ligand in the second compound. Anion **1** ^2−^ is [Mo(tfd)_2_(ox)]^2−^ and anion **2** ^2−^, formally generated by adding a Mo(tfd)_2_ fragment onto **1** ^2−^, is [(tfd)_2_Mo(μ-ox)Mo(tfd)_2_]^2−^. The crystalline material containing **1** ^2−^ is (N^*n*^Bu_4_)_2_-**1**·CHCl_3_·oxH_2_, while the material containing **2** ^2−^ is (N^*n*^Bu_4_)_2_-**2**. Anion **2** ^2−^ lies across an inversion centre. The complex anions afford a rare opportunity to compare terminal oxalate with bridging oxalate, coordinated to the same metal fragment, here (tfd)_2_Mo^IV^. C—O bond-length alternation is observed for the terminal oxalate ligand in **1** ^2−^: the difference between the C—O bond length involving the metal-coordinating O atom and the C—O bond length involving the uncoordinating O atom is 0.044 (12) Å. This bond-length alternation is significant but is smaller than the bond-length alternation observed for oxalic acid in the co-crystallized oxalic acid in (N^*n*^Bu_4_)_2_-**1**·CHCl_3_·oxH_2_, where a difference (for C=O *versus* C—OH) of 0.117 (14) Å was observed. In the bridging oxalate ligand in **2** ^2−^, the C—O bond lengths are equalized, within the error margin of one bond-length determination (0.006 Å). It is concluded that oxalic acid contains a localized π-system in its carb­oxy­lic acid groups, that the bridging oxalate ligand in **2** ^2−^ contains a delocalized π-system and that the terminal oxalate ligand in **1** ^2−^ contains an only partially localized π-system. In (N^*n*^Bu_4_)_2_-**1**·CHCl_3_·oxH_2_, the F atoms of two of the –CF_3_ groups in **1** ^2−^ are disordered over two sets of sites, as are the N and eight of the C atoms of one of the N^*n*^Bu_4_ cations. In (N^*n*^Bu_4_)_2_-**2**, the whole of the unique N^*n*^Bu_4_^+^ cation is disordered over two sets of sites. Also, in (N^*n*^Bu_4_)_2_-**2**, a region of disordered electron density was treated with the SQUEEZE routine in *PLATON* [Spek (2015 ▸). *Acta Cryst.* C**71**, 9–18].

## Abstract 2

[Mo(tfd)_2_(ox)]^2−^ as tetra-*n*-butyl­ammonium salt [co-crystal with oxalic acid and chloro­form; tfd is S_2_C_2_(CF_3_)_2_ and ox is C_2_O_4_] and [(tfd)_2_Mo(μ-ox)Mo(tfd)_2_]^2−^ as tetra-*n*-butyl­ammonium salt.

## Chemical context   

The oxalate (ox^2−^, C_2_O_4_^2−^) ion is a very useful ligand in transition metal chemistry. Its usefulness stems in part from its ability to act as a chelate ligand toward a metal cation while retaining two more O atoms with the ability to donate to another metal cation. Thus, while coordination compounds containing terminal oxalate are known, oxalates can easily act as bridging ligands to allow for the synthesis of dimetallic and multimetallic mol­ecular compounds, as well as extended coordination polymers (Clemente-León *et al.*, 2011 ▸; Gruselle *et al.*, 2006 ▸). Most of the work has involved V, Cr, Mn, Fe, Co, Ni and Cu, as well as Ru and Rh. Compounds where oxalate coordinates to molybdenum are rare, although some examples have been synthesized, mostly in the context of nitro­genase models, where oxalate was deemed a model for homocitrate (Demadis & Coucouvanis, 1995 ▸). Stimulated by our previous results on the molybdenum(IV) di­thiol­ene fragment Mo(tfd)_2_ [tfd^2−^ = S_2_C_2_(CF_3_)_2_^2−^] with a labile ‘cap’ (Harrison *et al.*, 2007 ▸; Nguyen *et al.*, 2010 ▸), we added oxalate to the Mo(tfd)_2_ fragment, as described in the ‘*Synthesis and crystallization*’ section (§5). The [Mo(tfd)_2_(ox)]^2−^ (**1** ^2−^) and [(tfd)_2_Mo(μ-ox)Mo(tfd)_2_]^2−^ (**2** ^2−^) anions were indeed obtained, offering an opportunity for a structural comparison.

## Structural commentary   

**Figure 1:**
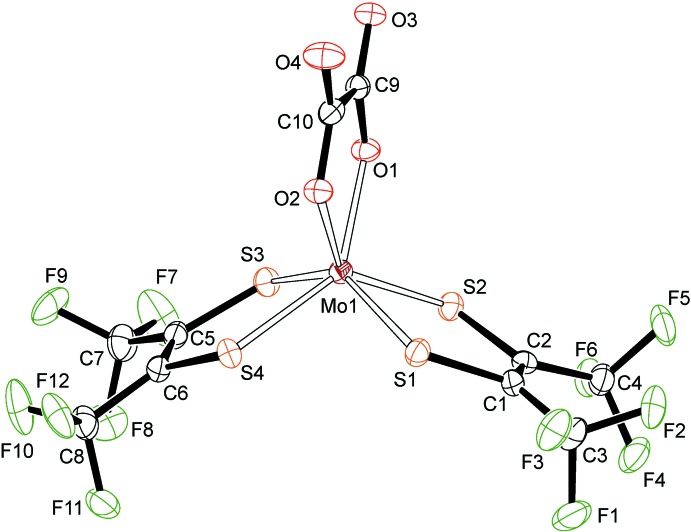
A view of the mol­ecular structure of **1** ^2−^ in (N^*n*^Bu_4_)_2_-**1**·CHCl_3_·oxH_2_. Anisotropic displacement ellipsoids are shown at the 30% probability level.

**Figure 2:**
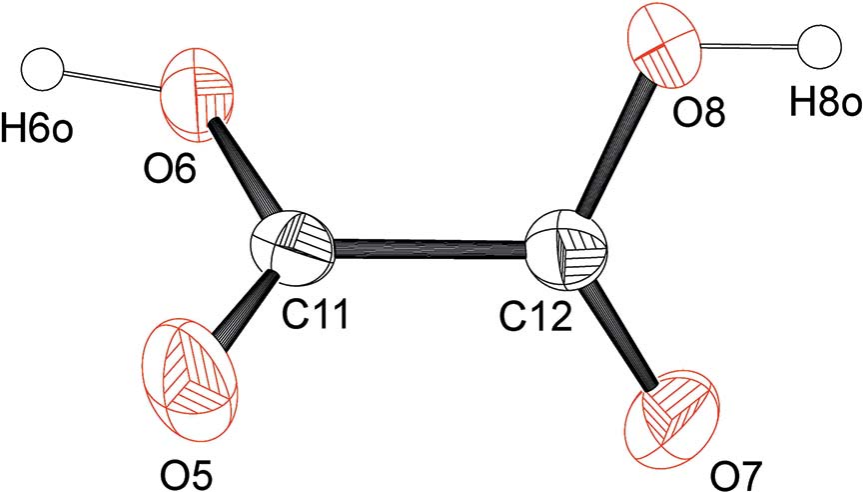
A view of the mol­ecular structure of the oxalic acid (oxH_2_) mol­ecule in (N^*n*^Bu_4_)_2_-**1**·CHCl_3_·oxH_2_. Anisotropic displacement ellipsoids are shown at the 30% probability level.

**Figure 3:**
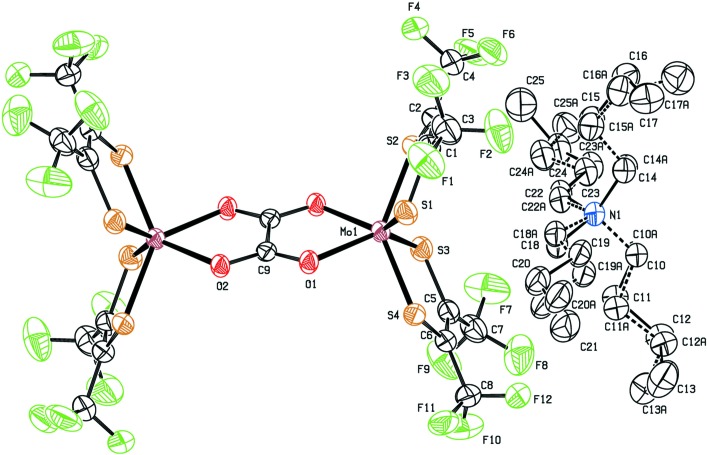
A view showing the **2** ^2−^ anion and the (disordered) N^*n*^Bu_4_^+^ cation in (N^*n*^Bu_4_)_2_-**2**. Anisotropic displacement ellipsoids are shown at the 30% probability level. The minor component of disorder is shown with dashed bonds. Unlabelled atoms are related by a crystallographic inversion centre (symmetry code: −*x* + 2, −*y*, −*z* + 1).

**Figure 4:**
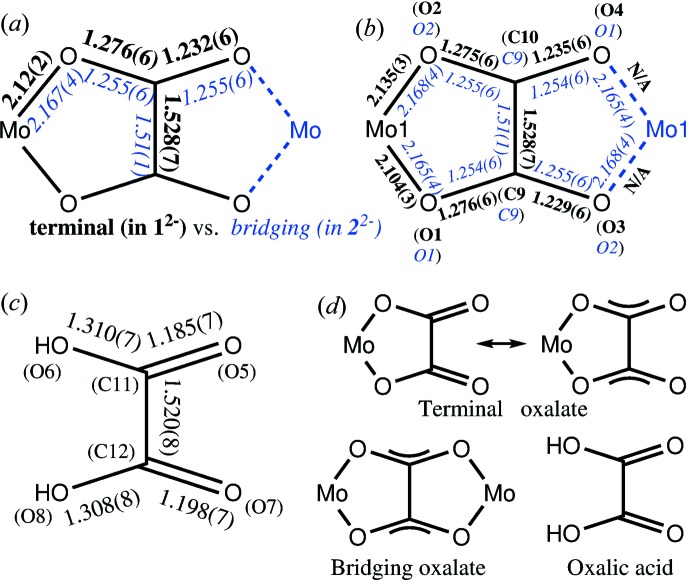
Bond-length changes on going from terminal to bridging oxalate, summarized (*a*) and in detail (*b*), as well as bond lengths in the oxalic acid mol­ecule observed (*c*) and concluding resonance description (*d*).

The counter-cation for both complex molybdate anions was tetra-*n*-butyl­ammonium. **1** ^2−^ was obtained as (N^*n*^Bu_4_)_2_-**1**·CHCl_3_·oxH_2_, while **2** ^2−^ was obtained as (N^*n*^Bu_4_)_2_-**2**. The mol­ecular structure of **1** ^2−^ is shown in Fig. 1 ▸, where N^*n*^Bu_4_^+^ counter-ions and co-crystallized oxalic acid, as well as chloro­form solvent mol­ecules, are not shown. Only one orientiation is shown for the disordered tri­fluoro­methyl groups involving atoms C7 and C8. The charge on the molybdenum-containing moiety, which is identified as **1** ^2−^, is unambiguous, due to the tetra-*n*-butyl­ammonium cations. While tfd can be redox-non-innocent (Hosking *et al.*, 2009 ▸), it is redox-innocent here. The C—C bond lengths in the two tfd ligand backbones [1.349 (8) Å for C1—C2 and 1.353 (8) Å for C5—C6] are a clear indication of fully reduced (dianionic) ene–di­thiol­ate (tfd^2−^), such that the oxidation state of the metal is +IV. The Mo—S bond lengths, ranging from 2.3265 (14) to 2.3390 (15) Å, are as expected for tfd complexes of Mo^IV^ (Nguyen *et al.*, 2010 ▸). Regarding the bonded oxalate, the average Mo—O bond length is 2.12 Å [Mo1—O1 = 2.104 (3) Å and Mo1—O2 = 2.135 (3) Å]. Within the oxalate unit, the chemically distinct O atoms (coordinating to molybdenum *versus* uncoordinating) show different bond lengths to the directly bonded C atom. The C—O bond length involving the metal-coordinating O atom is 1.276 (6) Å (average of two values), with the C—O bond length involving the uncoordinating O atom is 1.232 (6) Å (average of two values), for a difference of 0.044 (12) Å. While it may be tempting to describe the longer C—O bond as a single bond and the shorter C—O bond as a double bond, such a description would not be fully accurate since the bond-length alternation is only partial and less pronounced than for oxalic acid. The oxalic acid (oxH_2_) mol­ecule found in the structure of (N^*n*^Bu_4_)_2_-**1**·CHCl_3_·oxH_2_ is shown in Fig. 2 ▸. This oxalic acid mol­ecule exhibits stronger bond-length alternation: a difference (for C=O *versus* C—OH) of 0.117 (14) Å is observed. For further comparison, the structure of **2** ^2−^, in (N^*n*^Bu_4_)_2_-**2**, is valuable. Both **2** ^2−^ and the (disordered) tetra-*n*-butyl­ammonium ion in the structure of (N^*n*^Bu_4_)_2_-**2** are shown in Fig. 3 ▸. For the bridging oxalate ligand in **2** ^2−^, bond-length equalization is observed, within the error margin of one bond-length determination (0.006 Å). The details of the oxalate substructure are shown in Fig. 4 ▸, where Fig. 4 ▸(*a*) highlights the bond-length changes on going from a terminal oxalate in **1** ^2−^ to a bridging oxalate in **2** ^2−^, where parameters related to chemically equivalent bonds are averaged for clarity, and Fig. 4 ▸(*b*) shows all data before averaging. Fig. 4 ▸(*c*) shows the bond lengths in the free oxalic acid mol­ecule in (N^*n*^Bu_4_)_2_-**1**·CHCl_3_·oxH_2_. Fig. 4 ▸(*d*) summarizes the findings: oxalic acid contains a localized π-system in its carb­oxy­lic acid groups, the bridging oxalate in **2** ^2−^ contains a delocalized π-system and terminal oxalate in **1** ^2−^ contains a partially localized π-system. While only marginally significant (*ca* 1σ), an effect involving the C—C bonds of oxalate can be seen: upon becoming bridging, the oxalate C—C bond shortens from 1.528 (7) Å to 1.51 (1) Å (Figs. 4 ▸ *a* and 4*b*). While this bond shortening may initially be surprising, it is actually theoretically expected: the π-system in a localized butadiene-like system is anti­bonding with respect to the central C—C bond. When oxalate becomes bridging, due to delocalization in the π-system, the electronic structure is no longer butadiene-like but rather resembles two allyl anions linked at the central C atom, where the π-overlap at the central C atoms is not anti­bonding but just nonbonding. Apart from the specifics of the oxalate substructure in **2** ^2−^, there are no dramatic changes in the coordination sphere of molybdenum on going from **1** ^2−^ to **2** ^2−^. The points made above for **1** ^2−^ related to Mo—S bond lengths (normal) and C—C bond lengths in the tfd ligand (double bond) typically apply also to **2** ^2−^. Also, both metal centres much more closely resemble a trigonal prismatic structure than an octa­hedral structure, as is expected for *d* ^2^ tris-chelates involving di­thiol­enes. Using the *X*—*M*—*X_trans_* criterion (Beswick *et al.*, 2004 ▸; Nguyen *et al.*, 2010 ▸), the geometry around molybdenum in **1** ^2−^ is 88% trigonal-prismatic. Using the same method, the geometry around molybdenum in **2** ^2−^ analyzes as 99% trigonal-prismatic.
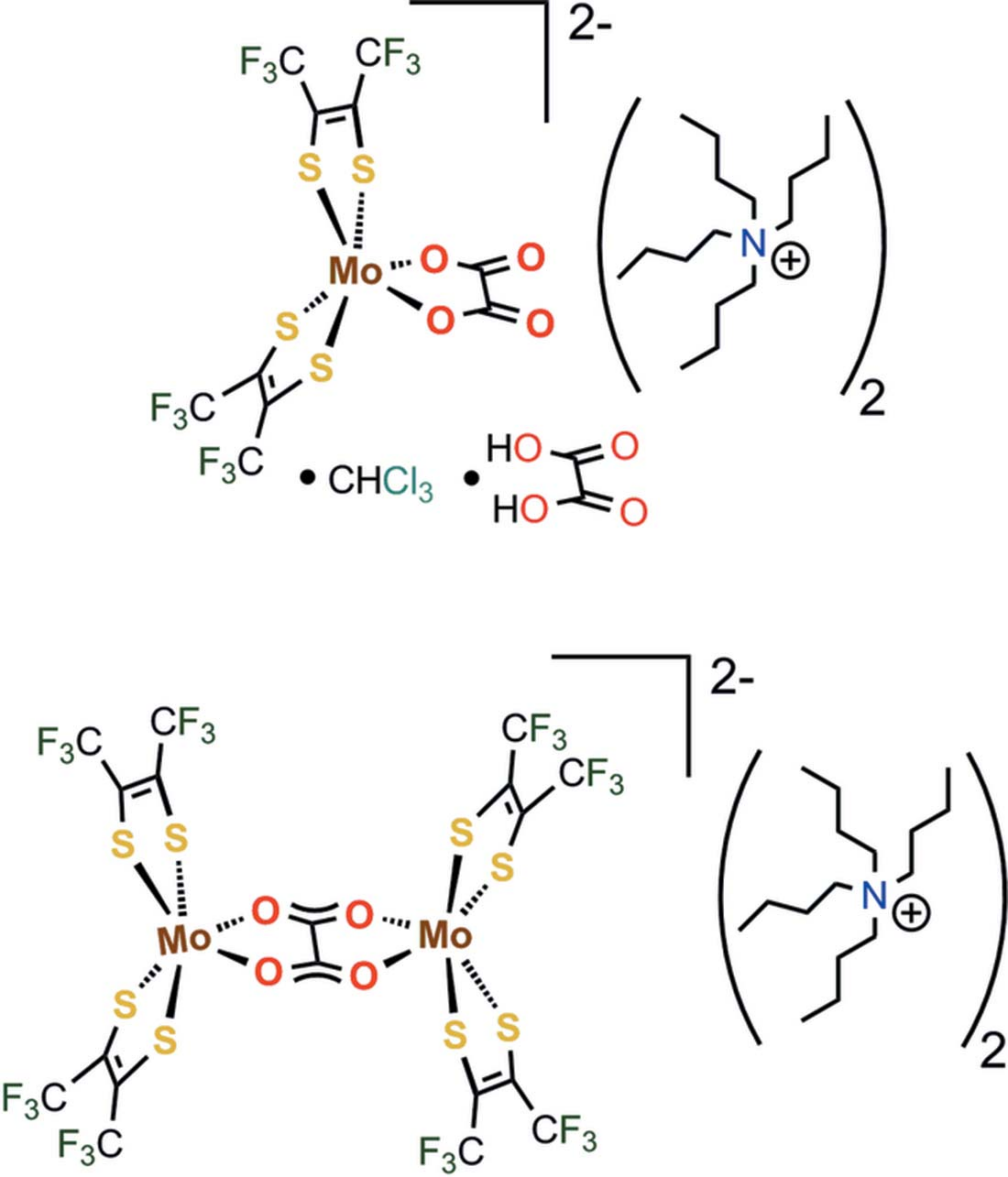


## Supra­molecular features   

**Figure 5:**
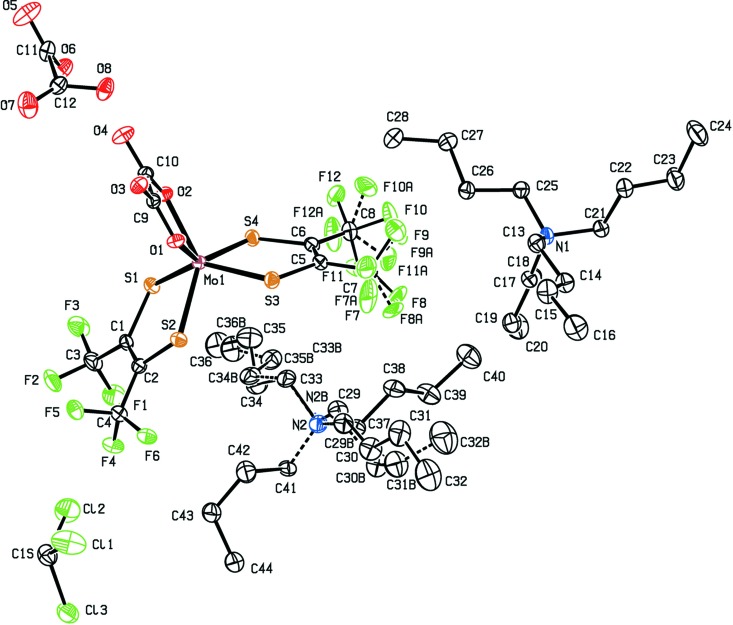
Anisotropic displacement plot (30% probability level) showing all non-H atoms (including disordered ones and those of chloro­form solvent) in (N^*n*^Bu_4_)_2_-**1**·CHCl_3_·oxH_2_. The minor component of disorder is shown with dashed bonds. Atom N2 is disordered over two sites and the major component is obscured by the minor component.

The oxalic acid solvent molecule and the metal-coordinating oxalate ligand in (N^*n*^Bu_4_)_2_-**1**·CHCl_3_·oxH_2_ form a hydrogen-bonded network (Table 1 ▸). The oxalate O atoms of **1** ^2−^ that are not metal coordinating act as hydrogen-bond acceptors. Oxalic acid acts as a hydrogen-bond donor: both of its OH functionalities hydrogen bond to two different mol­ecules of **1** ^2−^, such that infinite chains along [100] of the type ‘—**1** ^2−^—HOOC-COOH—**1** ^2−^—, *etc*’ are formed. The (N^*n*^Bu_4_)_2_^+^ cations (one of them containing disorder) are packed around the **1** ^2−^ anion, along with a CHCl_3_ solvent mol­ecule that forms part of the structure. A plot showing anisotropic displacement ellipsoids for all non-H atoms (including disordered ones) in (N^*n*^Bu_4_)_2_-**1**·CHCl_3_·oxH_2_ is shown in Fig. 5 ▸. In contrast, there are no hydrogen bonds or notable close contacts in the structure of (N^*n*^Bu_4_)_2_-**2**, which consists of a packing of **2** ^2−^ anions and N^*n*^Bu_4_^+^ cations, both of which are shown in Fig. 3 ▸.

## Database survey   

Relevant coordination compounds containing di­thiol­enes are discussed above, where review articles for coordinating oxalate are also referenced. A search of the Cambridge Structural Database (Version 5.38, including updates up to May 2017; Groom *et al.*, 2016 ▸) reveals no reports of molybdenum dithiolene complexes that contain oxalate.

## Synthesis and crystallization   

### General specifications   

All manipulations involving metal-containing compounds were carried out under an inert (N_2_) atmosphere using standard glove-box (M. Braun UniLab) and Schlenk techniques. Solvents were purified prior to use by vacuum distillation from mol­ecular sieves. Organic and inorganic starting materials were obtained from Sigma–Aldrich. Mo(tfd)_2_(tht)_2_ (tht = tetra­hydro­thio­phene) was synthesized from Mo(tfd)_2_(bdt) (bdt = S_2_C_6_H_4_) as in Nguyen *et al.* (2010 ▸). Mo(tfd)_2_(bdt) was synthesized as in Harrison *et al.* (2007 ▸). Tetra-*n*-butyl­ammonium oxalate was prepared by neutralizing oxalic acid with aqueous tetra­butyl­ammonium hydroxide, followed by drying under vacuum at 333 K.

### Synthesis of (N^*n*^Bu_4_)_2_-**1**.CHCl_3_·oxH_2_   

We were unable to obtain **1** ^2−^ as the only molybdenum product produced in a reaction. Attempts always led to significant decomposition to form a blue material, almost certainly molybdenum that is reduced below the oxidation state +IV due to the reducing power of oxalate. However, **1** ^2−^ can be obtained as crystals (co-crystals with oxalic acid and chloro­form) in the form of brown blocks. 2 mg of Mo(tfd)_2_(bdt) (2.9 µmol) were dissolved in a small amount of chloro­form in a glass vial. In a second glass vial, 16.7 mg (29 µmol) of tetra-*n*-butyl­ammonium oxalate were dissolved in the amount of chloro­form needed to create a clear solution. The contents of the two vials were mixed and 3.3 µl (14.6 µmol) of bis­(tri­methyl­sil­yl)acetyl­ene, needed to labilize the bdt fragment (Nguyen *et al.*, 2010 ▸), were added *via* microlitre syringe. The initially dark (blue–green) solution became lighter, and small brown particles began to form. After 72 h, the solvent was reduced under vacuum, and orange–brown crystals grew. Blue–green needles (not of X-ray quality) of a different (likely reduced) molybdenum product were also growing. The orange–brown blocks were manually separated and chosen for X-ray crystallography.

### Synthesis of (N^*n*^Bu_4_)_2_-**2**   

2 mg (2.8 µmol) of Mo(tfd)_2_(tht)_2_ were dissolved in a minimal amount of chloro­form. A solution of 16 mg (28 µmol) of tetra-*n*-butyl­ammonium oxalate in 2 ml of chloro­form was added. The solution turned red and, after 2 h, thin pink rectangular crystals had formed. The liquid was deca­nted and the crystals were washed twice with chloro­form and dried under vacuum. X-ray-quality crystals were grown using vapour diffusion. In a small vial, the product was dissolved in di­chloro­methane. The small vial was placed uncapped into a larger vial with chloro­form. The larger vial was capped, and over a period of 2 d, the di­chloro­methane solvent had evaporated from the small vial and dissolved in the chloro­form in the larger vial, leaving pink crystals in the smaller vial. The crystals were found to be very air-sensitive, and exposure to air leads to decomposition to form a liquid that colours the surface of the crystals initially green and later blue.

## Refinement   

Crystal data, data collection and structure refinement details are summarized in Table 2 ▸. In (N^*n*^Bu_4_)_2_-**1**·CHCl_3_·oxH_2_, H atoms bonded to C atoms were placed in calculated positions and included in a riding-motion approximation, while H atoms bonded to O atoms were refined independently with isotropic displacement parameters. In the anion **1** ^2−^, atoms F7/F8/F9 were included as disordered over two sets of sites, with refined occupancies of 0.58 (2) and 0.42 (2). Atoms F10/F11/F12 were included as disordered, with refined occupancies of 0.502 (10) and 0.498 (10). The C—F bond lengths and F⋯F distances were restrained using the SADI command in *SHELXL* (Sheldrick, 2015 ▸) and the anisotropic displacement parameters of the disordered F atoms and bonded C atoms were restrained using the SIMU command. In addition, the N and 8 C atoms (C29–C36) of one of the independent N^*n*^Bu_4_^+^ cations were refined as disordered over two sets of sites, with refined occupancies of 0.676 (9) and 0.324 (9). The SAME command in *SHELXL* was used to restrain the geometry of the disordered C-atom chains to those of the ordered N^*n*^Bu_4_^+^ cation and the SIMU command was used to restrain anisotropic displacement parameters of the disordered atoms. In (N^*n*^Bu_4_)_2_-**2**, all H atoms were placed in calculated positions and refined in a riding-motion approximation. During the refinement of the structure of (N^*n*^Bu_4_)_2_-**2**, electron-density peaks were located that were believed to be highly disordered solvent mol­ecules (crystallization solvents were CH_2_Cl_2_/CHCl_3_). Attempts made to model the solvent mol­ecule were not successful. The SQUEEZE (Spek, 2015 ▸) option in *PLATON* (Spek, 2009 ▸) indicated that there was a large solvent cavity of 156 Å. In the final cycles of refinement, this contribution of 62.6 electrons to the electron density was removed from the observed data. The density, the *F*(000) value, the mol­ecular weight and the formula are given without taking into account the results obtained with the SQUEEZE option. Similar treatments of disordered solvent mol­ecules were carried out by Stähler *et al.* (2001 ▸), Cox *et al.* (2003 ▸), Mohamed *et al.* (2003 ▸) and Athimoolam *et al.* (2005 ▸). Also in (N^*n*^Bu_4_)_2_-**2**, the whole mol­ecule of the unique N^*n*^Bu_4_^+^ cation was included as disordered over two sets of sites, with refined occupancies of 0.589 (6) and 0.411 (6). The same command in *SHELXL* was used to restrain the geometry of the minor component of disorder to that of the major component and the SIMU command was used to restrain all anisotropic diplacement parameters of the disordered atoms.

## Supplementary Material

Crystal structure: contains datablock(s) k10131, k10171_sq. DOI: 10.1107/S205698901701026X/zl2708sup1.cif

Structure factors: contains datablock(s) k10131. DOI: 10.1107/S205698901701026X/zl2708k10131sup2.hkl

Structure factors: contains datablock(s) k10171_sq. DOI: 10.1107/S205698901701026X/zl2708k10171_sqsup3.hkl

CCDC references: 1561379, 1561378

Additional supporting information:  crystallographic information; 3D view; checkCIF report

## Figures and Tables

**Table 1:** Hydrogen-bond geometry (Å, °) for (N^*n*^Bu_4_)_2_-**1**·CHCl_3_·oxH_2_ [Chem scheme1]

*D*—H⋯*A*	*D*—H	H⋯*A*	*D*⋯*A*	*D*—H⋯*A*
O6—H6O⋯O3^i^	0.88 (7)	1.76 (7)	2.633 (5)	170 (7)
O8—H8O⋯O4	0.85 (8)	1.75 (8)	2.587 (5)	174 (9)

Symmetry code: (i) 
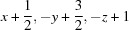
.

**Table 2:** Experimental details

	(N^*n*^Bu_4_)_2_-**1**·CHCl_3_·C_2_H_2_O_4_	(N^*n*^Bu_4_)_2_-**2**
Crystal data		
Chemical formula	(C_16_H_36_N)_2_[Mo(C_4_F_6_S_2_)_2_(C_2_O_4_)]·C_2_H_2_O_4_·CHCl_3_	(C_16_H_36_N)[Mo_2_(C_4_F_6_S_2_)_4_(C_2_O_4_)]
*M*_r_	1330.60	1669.45
Crystal system, space group	Orthorhombic, *P*2_1_2_1_2_1_	Monoclinic, *P*2_1_/*n*
Temperature (K)	150	150
*a*, *b*, *c* (Å)	15.3879 (2), 17.8733 (5), 22.2895 (6)	14.2347 (15), 19.4940 (19), 14.4056 (14)
α, β, γ (°)	90, 90, 90	90, 103.159 (5), 90
*V* (Å^3^)	6130.3 (3)	3892.5 (7)
*Z*	4	2
Radiation type	Mo *K*α	Mo *K*α
μ (mm^−1^)	0.56	0.63
Crystal size (mm)	0.15 × 0.12 × 0.10	0.18 × 0.18 × 0.06

Data collection		
Diffractometer	Nonius KappaCCD	Nonius KappaCCD
Absorption correction	Multi-scan (*SORTAV*; Blessing, 1995 ▸)	Multi-scan (*SORTAV*; Blessing, 1995 ▸)
*T*_min_, *T*_max_	0.759, 0.869	0.720, 0.931
No. of measured, independent and observed [*I* > 2σ(*I*)] reflections	40314, 13841, 9858	18527, 7278, 4243
*R*_int_	0.065	0.066
(sin θ/λ)_max_ (Å^−1^)	0.650	0.613

Refinement		
*R*[*F* ^2^ > 2σ(*F* ^2^)], *wR*(*F* ^2^), *S*	0.048, 0.105, 1.02	0.065, 0.164, 1.01
No. of reflections	13841	7278
No. of parameters	816	560
No. of restraints	465	520
H-atom treatment	H atoms treated by a mixture of independent and constrained refinement	H-atom parameters constrained
Δρ_max_, Δρ_min_ (e Å^−3^)	0.61, −0.66	0.59, −0.65
Absolute structure	Flack *x* determined using 3456 quotients [(*I* ^+^) − (*I* ^−^)]/[(*I* ^+^) + (*I* ^−^)] (Parsons *et al.*, 2013 ▸)	–
Absolute structure parameter	−0.036 (18)	–

Computer programs: *COLLECT* (Nonius, 2002 ▸), *DENZO-SMN* (Otwinowski & Minor, 1997 ▸), *SIR92* (Altomare *et al.*, 1994 ▸), *SHELXL2014* (Sheldrick, 2015 ▸), *ORTEP-3* (Farrugia, 2012 ▸), *PLATON* (Spek, 2009 ▸) and *SHELXTL* (Sheldrick, 2008 ▸).

## supplementary crystallographic information

### Bis(tetra-n-butylammonium) bis(1,1,1,4,4,4-hexafluorobut-2-ene-2,3-dithiolato)oxalatomolybdate(IV)–chloroform–oxalic acid (1/1/1), (k10131) Crystal data

**Table:**

(C_16_H_36_N)_2_[Mo(C_4_F_6_S_2_)_2_(C_2_O_4_)]·C_2_H_2_O_4_·CHCl_3_	*D*_x_ = 1.442 Mg m^−^^3^
*M**_r_* = 1330.60	Mo *K*α radiation, λ = 0.71073 Å
Orthorhombic, *P*2_1_2_1_2_1_	Cell parameters from 31375 reflections
*a* = 15.3879 (2) Å	θ = 2.6–27.5°
*b* = 17.8733 (5) Å	µ = 0.56 mm^−^^1^
*c* = 22.2895 (6) Å	*T* = 150 K
*V* = 6130.3 (3) Å^3^	Block, brown
*Z* = 4	0.15 × 0.12 × 0.10 mm
*F*(000) = 2752	

### Bis(tetra-n-butylammonium) bis(1,1,1,4,4,4-hexafluorobut-2-ene-2,3-dithiolato)oxalatomolybdate(IV)–chloroform–oxalic acid (1/1/1), (k10131) Data collection

**Table:**

Nonius KappaCCD diffractometer	13841 independent reflections
Radiation source: fine-focus sealed tube	9858 reflections with *I* > 2σ(*I*)
Detector resolution: 9 pixels mm^-1^	*R*_int_ = 0.065
φ scans and ω scans with κ offsets	θ_max_ = 27.5°, θ_min_ = 2.6°
Absorption correction: multi-scan SORTAV (Blessing, 1995)	*h* = −19→19
*T*_min_ = 0.759, *T*_max_ = 0.869	*k* = −23→23
40314 measured reflections	*l* = −28→28

### Bis(tetra-n-butylammonium) bis(1,1,1,4,4,4-hexafluorobut-2-ene-2,3-dithiolato)oxalatomolybdate(IV)–chloroform–oxalic acid (1/1/1), (k10131) Refinement

**Table:**

Refinement on *F*^2^	Hydrogen site location: mixed
Least-squares matrix: full	H atoms treated by a mixture of independent and constrained refinement
*R*[*F*^2^ > 2σ(*F*^2^)] = 0.048	*w* = 1/[σ^2^(*F*_o_^2^) + (0.0407*P*)^2^ + 1.6697*P*] where *P* = (*F*_o_^2^ + 2*F*_c_^2^)/3
*wR*(*F*^2^) = 0.105	(Δ/σ)_max_ = 0.001
*S* = 1.02	Δρ_max_ = 0.61 e Å^−^^3^
13841 reflections	Δρ_min_ = −0.66 e Å^−^^3^
816 parameters	Absolute structure: Flack *x* determined using 3456 quotients [(I+)-(I-)]/[(I+)+(I-)] (Parsons *et al.*, 2013)
465 restraints	Absolute structure parameter: −0.036 (18)

### Bis(tetra-n-butylammonium) bis(1,1,1,4,4,4-hexafluorobut-2-ene-2,3-dithiolato)oxalatomolybdate(IV)–chloroform–oxalic acid (1/1/1), (k10131) Special details

**Table:**

Geometry. All esds (except the esd in the dihedral angle between two l.s. planes) are estimated using the full covariance matrix. The cell esds are taken into account individually in the estimation of esds in distances, angles and torsion angles; correlations between esds in cell parameters are only used when they are defined by crystal symmetry. An approximate (isotropic) treatment of cell esds is used for estimating esds involving l.s. planes.

### Bis(tetra-n-butylammonium) bis(1,1,1,4,4,4-hexafluorobut-2-ene-2,3-dithiolato)oxalatomolybdate(IV)–chloroform–oxalic acid (1/1/1), (k10131) Fractional atomic coordinates and isotropic or equivalent isotropic displacement parameters (Å^2^)

**Table:**

	*x*	*y*	*z*	*U*_iso_*/*U*_eq_	Occ. (<1)
Mo1	0.53960 (3)	0.43946 (2)	0.51037 (2)	0.02584 (12)	
S1	0.66590 (8)	0.46380 (8)	0.45643 (7)	0.0323 (3)	
S2	0.52665 (9)	0.34333 (8)	0.44027 (7)	0.0337 (3)	
S3	0.47571 (9)	0.35062 (8)	0.57355 (7)	0.0340 (3)	
S4	0.63308 (8)	0.45238 (8)	0.59206 (6)	0.0322 (3)	
F1	0.8097 (2)	0.3630 (2)	0.3621 (2)	0.0635 (12)	
F2	0.7261 (3)	0.4127 (2)	0.29583 (17)	0.0606 (11)	
F3	0.7876 (2)	0.4809 (2)	0.36285 (18)	0.0591 (11)	
F4	0.6817 (3)	0.2702 (2)	0.31926 (18)	0.0577 (10)	
F5	0.5674 (3)	0.3269 (2)	0.28819 (16)	0.0606 (11)	
F6	0.5567 (2)	0.23584 (19)	0.34988 (16)	0.0507 (9)	
F7	0.4324 (9)	0.2510 (8)	0.6659 (5)	0.091 (4)	0.58 (2)
F8	0.5560 (6)	0.2515 (6)	0.7082 (6)	0.062 (3)	0.58 (2)
F9	0.4584 (10)	0.3280 (6)	0.7376 (4)	0.066 (3)	0.58 (2)
F7A	0.4066 (4)	0.2880 (9)	0.6787 (6)	0.061 (4)	0.42 (2)
F8A	0.5269 (10)	0.2300 (5)	0.6931 (7)	0.061 (4)	0.42 (2)
F9A	0.4962 (10)	0.3283 (8)	0.7436 (4)	0.054 (4)	0.42 (2)
F10	0.6162 (7)	0.3786 (8)	0.7546 (4)	0.083 (4)	0.502 (10)
F11	0.7244 (7)	0.3497 (7)	0.6994 (5)	0.079 (4)	0.502 (10)
F12	0.6920 (8)	0.4619 (5)	0.7150 (5)	0.061 (3)	0.502 (10)
F10A	0.6267 (8)	0.4412 (7)	0.7472 (4)	0.084 (4)	0.498 (10)
F11A	0.6637 (7)	0.3297 (5)	0.7316 (4)	0.059 (3)	0.498 (10)
F12A	0.7358 (7)	0.4176 (9)	0.6927 (5)	0.084 (4)	0.498 (10)
O1	0.4106 (2)	0.46339 (18)	0.48554 (18)	0.0313 (8)	
O2	0.52764 (19)	0.55847 (17)	0.51231 (16)	0.0296 (7)	
O3	0.3096 (2)	0.5499 (2)	0.46934 (17)	0.0339 (9)	
O4	0.4395 (2)	0.65521 (19)	0.4943 (2)	0.0452 (11)	
C1	0.6717 (3)	0.4035 (3)	0.3947 (3)	0.0335 (13)	
C2	0.6103 (3)	0.3501 (3)	0.3889 (3)	0.0313 (13)	
C3	0.7484 (4)	0.4144 (4)	0.3540 (3)	0.0480 (17)	
C4	0.6037 (4)	0.2963 (4)	0.3374 (3)	0.0423 (15)	
C5	0.5288 (4)	0.3503 (3)	0.6422 (3)	0.0341 (13)	
C6	0.5997 (3)	0.3938 (3)	0.6504 (3)	0.0335 (13)	
C7	0.4924 (3)	0.2977 (3)	0.6884 (3)	0.0517 (17)	
C8	0.6569 (4)	0.3958 (4)	0.7050 (3)	0.0452 (15)	
C9	0.3838 (3)	0.5309 (3)	0.4828 (3)	0.0282 (12)	
C10	0.4552 (3)	0.5876 (3)	0.4974 (2)	0.0305 (12)	
O5	0.6031 (3)	0.9245 (2)	0.4773 (3)	0.0739 (17)	
O6	0.7088 (3)	0.8409 (2)	0.4927 (2)	0.0486 (12)	
H6O	0.748 (4)	0.874 (4)	0.504 (4)	0.07 (2)*	
O7	0.5404 (3)	0.7847 (3)	0.4176 (2)	0.0660 (13)	
O8	0.5587 (3)	0.7544 (2)	0.5137 (2)	0.0496 (12)	
H8O	0.521 (5)	0.720 (4)	0.509 (4)	0.09 (3)*	
C11	0.6297 (4)	0.8623 (3)	0.4796 (3)	0.0411 (15)	
C12	0.5716 (4)	0.7956 (3)	0.4660 (3)	0.0392 (15)	
N1	0.2406 (3)	0.1717 (2)	0.8798 (2)	0.0330 (11)	
C13	0.1512 (3)	0.1525 (3)	0.8555 (3)	0.0403 (15)	
H13A	0.1077	0.1834	0.8769	0.048*	
H13B	0.1492	0.1671	0.8126	0.048*	
C14	0.1240 (3)	0.0719 (3)	0.8603 (3)	0.0450 (16)	
H14A	0.1172	0.0585	0.9031	0.054*	
H14B	0.1701	0.0397	0.8431	0.054*	
C15	0.0400 (4)	0.0572 (4)	0.8279 (3)	0.0608 (18)	
H15A	−0.0041	0.0930	0.8428	0.073*	
H15B	0.0489	0.0677	0.7847	0.073*	
C16	0.0044 (5)	−0.0205 (4)	0.8341 (4)	0.075 (2)	
H16A	−0.0501	−0.0245	0.8116	0.113*	
H16B	0.0465	−0.0565	0.8183	0.113*	
H16C	−0.0066	−0.0312	0.8766	0.113*	
C17	0.3091 (3)	0.1358 (3)	0.8406 (3)	0.0373 (14)	
H17A	0.3007	0.0809	0.8423	0.045*	
H17B	0.2982	0.1515	0.7986	0.045*	
C18	0.4029 (4)	0.1520 (3)	0.8548 (3)	0.0434 (15)	
H18A	0.4169	0.1333	0.8955	0.052*	
H18B	0.4129	0.2067	0.8542	0.052*	
C19	0.4612 (4)	0.1146 (3)	0.8095 (3)	0.0482 (16)	
H19A	0.4485	0.1356	0.7693	0.058*	
H19B	0.4472	0.0606	0.8083	0.058*	
C20	0.5571 (4)	0.1237 (5)	0.8221 (4)	0.073 (2)	
H20A	0.5907	0.0981	0.7909	0.109*	
H20B	0.5720	0.1770	0.8223	0.109*	
H20C	0.5707	0.1019	0.8613	0.109*	
C21	0.2504 (4)	0.1446 (3)	0.9443 (3)	0.0390 (14)	
H21A	0.2486	0.0893	0.9441	0.047*	
H21B	0.3086	0.1597	0.9588	0.047*	
C22	0.1836 (4)	0.1725 (4)	0.9886 (3)	0.0456 (15)	
H22A	0.1784	0.2275	0.9849	0.055*	
H22B	0.1264	0.1503	0.9788	0.055*	
C23	0.2072 (5)	0.1529 (5)	1.0519 (3)	0.071 (2)	
H23A	0.2687	0.1668	1.0590	0.085*	
H23B	0.2021	0.0981	1.0572	0.085*	
C24	0.1512 (6)	0.1912 (5)	1.0982 (4)	0.090 (3)	
H24A	0.1699	0.1761	1.1384	0.135*	
H24B	0.1570	0.2456	1.0940	0.135*	
H24C	0.0904	0.1768	1.0921	0.135*	
C25	0.2505 (4)	0.2567 (3)	0.8801 (3)	0.0383 (15)	
H25A	0.2009	0.2779	0.9027	0.046*	
H25B	0.3039	0.2690	0.9028	0.046*	
C26	0.2553 (4)	0.2964 (3)	0.8206 (3)	0.0393 (14)	
H26A	0.2022	0.2856	0.7971	0.047*	
H26B	0.3059	0.2778	0.7976	0.047*	
C27	0.2637 (5)	0.3796 (3)	0.8299 (3)	0.0517 (18)	
H27A	0.2145	0.3970	0.8549	0.062*	
H27B	0.3180	0.3897	0.8523	0.062*	
C28	0.2649 (5)	0.4242 (3)	0.7727 (3)	0.061 (2)	
H28A	0.2704	0.4775	0.7823	0.092*	
H28B	0.3142	0.4084	0.7480	0.092*	
H28C	0.2107	0.4157	0.7507	0.092*	
N2	0.7148 (8)	0.1623 (6)	0.5600 (15)	0.040 (2)	0.676 (9)
C29	0.6227 (6)	0.1461 (5)	0.5812 (8)	0.047 (2)	0.676 (9)
H29A	0.6124	0.1738	0.6189	0.056*	0.676 (9)
H29B	0.5815	0.1656	0.5509	0.056*	0.676 (9)
C30	0.6027 (6)	0.0649 (6)	0.5918 (7)	0.059 (3)	0.676 (9)
H30A	0.6512	0.0424	0.6147	0.071*	0.676 (9)
H30B	0.5996	0.0392	0.5525	0.071*	0.676 (9)
C31	0.5193 (6)	0.0509 (6)	0.6252 (6)	0.068 (3)	0.676 (9)
H31A	0.4712	0.0771	0.6044	0.082*	0.676 (9)
H31B	0.5241	0.0721	0.6661	0.082*	0.676 (9)
C32	0.4978 (8)	−0.0321 (6)	0.6296 (7)	0.093 (4)	0.676 (9)
H32A	0.4431	−0.0385	0.6517	0.139*	0.676 (9)
H32B	0.4917	−0.0531	0.5892	0.139*	0.676 (9)
H32C	0.5446	−0.0581	0.6509	0.139*	0.676 (9)
C33	0.7243 (6)	0.2474 (5)	0.5539 (6)	0.052 (2)	0.676 (9)
H33A	0.6747	0.2663	0.5300	0.062*	0.676 (9)
H33B	0.7197	0.2698	0.5944	0.062*	0.676 (9)
C34	0.8069 (8)	0.2748 (5)	0.5254 (6)	0.060 (3)	0.676 (9)
H34A	0.8536	0.2380	0.5332	0.072*	0.676 (9)
H34B	0.7984	0.2777	0.4814	0.072*	0.676 (9)
C35	0.8359 (6)	0.3499 (6)	0.5477 (6)	0.067 (3)	0.676 (9)
H35A	0.8475	0.3466	0.5913	0.081*	0.676 (9)
H35B	0.7883	0.3865	0.5417	0.081*	0.676 (9)
C36	0.9162 (9)	0.3777 (9)	0.5164 (7)	0.075 (3)	0.676 (9)
H36A	0.9324	0.4267	0.5326	0.112*	0.676 (9)
H36B	0.9639	0.3423	0.5230	0.112*	0.676 (9)
H36C	0.9047	0.3821	0.4733	0.112*	0.676 (9)
N2B	0.7037 (17)	0.1560 (12)	0.559 (3)	0.043 (3)	0.324 (9)
C29B	0.6174 (13)	0.1228 (10)	0.5788 (19)	0.047 (4)	0.324 (9)
H29C	0.5987	0.1487	0.6159	0.056*	0.324 (9)
H29D	0.5737	0.1338	0.5475	0.056*	0.324 (9)
C30B	0.6165 (12)	0.0401 (10)	0.5906 (15)	0.059 (4)	0.324 (9)
H30C	0.6546	0.0290	0.6252	0.071*	0.324 (9)
H30D	0.6402	0.0136	0.5552	0.071*	0.324 (9)
C31B	0.5264 (13)	0.0112 (13)	0.6035 (10)	0.073 (4)	0.324 (9)
H31C	0.5213	−0.0403	0.5875	0.088*	0.324 (9)
H31D	0.4837	0.0428	0.5821	0.088*	0.324 (9)
C32B	0.5037 (18)	0.010 (2)	0.6694 (11)	0.115 (8)	0.324 (9)
H32D	0.4445	−0.0090	0.6746	0.173*	0.324 (9)
H32E	0.5447	−0.0220	0.6909	0.173*	0.324 (9)
H32F	0.5071	0.0612	0.6855	0.173*	0.324 (9)
C33B	0.6899 (11)	0.2402 (9)	0.5501 (12)	0.044 (3)	0.324 (9)
H33C	0.6355	0.2471	0.5269	0.053*	0.324 (9)
H33D	0.6807	0.2632	0.5900	0.053*	0.324 (9)
C34B	0.7607 (11)	0.2829 (10)	0.5188 (8)	0.048 (3)	0.324 (9)
H34C	0.7356	0.3291	0.5015	0.058*	0.324 (9)
H34D	0.7831	0.2522	0.4852	0.058*	0.324 (9)
C35B	0.8356 (10)	0.3040 (14)	0.5587 (9)	0.053 (4)	0.324 (9)
H35C	0.8678	0.2582	0.5701	0.063*	0.324 (9)
H35D	0.8127	0.3269	0.5960	0.063*	0.324 (9)
C36B	0.897 (2)	0.358 (2)	0.5291 (15)	0.075 (3)	0.324 (9)
H36D	0.9447	0.3700	0.5568	0.112*	0.324 (9)
H36E	0.9211	0.3352	0.4927	0.112*	0.324 (9)
H36F	0.8661	0.4039	0.5185	0.112*	0.324 (9)
C37	0.7786 (4)	0.1361 (3)	0.6058 (3)	0.0390 (15)	
H37A	0.8371	0.1538	0.5938	0.047*	
H37B	0.7798	0.0807	0.6051	0.047*	
C38	0.7617 (4)	0.1612 (4)	0.6693 (3)	0.0515 (18)	
H38A	0.7524	0.2160	0.6698	0.062*	
H38B	0.7080	0.1370	0.6842	0.062*	
C39	0.8358 (4)	0.1420 (4)	0.7105 (3)	0.059 (2)	
H39A	0.8887	0.1683	0.6966	0.070*	
H39B	0.8472	0.0876	0.7080	0.070*	
C40	0.8188 (5)	0.1628 (5)	0.7757 (4)	0.072 (2)	
H40A	0.8693	0.1490	0.8001	0.107*	
H40B	0.8089	0.2168	0.7787	0.107*	
H40C	0.7674	0.1360	0.7901	0.107*	
C41	0.7343 (4)	0.1213 (3)	0.5013 (3)	0.0406 (15)	
H41A	0.7941	0.1346	0.4889	0.049*	
H41B	0.7338	0.0669	0.5097	0.049*	
C42	0.6744 (4)	0.1355 (4)	0.4483 (3)	0.058 (2)	
H42A	0.6192	0.1081	0.4544	0.069*	
H42B	0.6607	0.1896	0.4461	0.069*	
C43	0.7156 (5)	0.1107 (4)	0.3897 (3)	0.0543 (18)	
H43A	0.6733	0.1189	0.3569	0.065*	
H43B	0.7665	0.1431	0.3816	0.065*	
C44	0.7451 (5)	0.0299 (4)	0.3876 (3)	0.062 (2)	
H44A	0.7707	0.0192	0.3482	0.093*	
H44B	0.7886	0.0212	0.4189	0.093*	
H44C	0.6952	−0.0031	0.3942	0.093*	
Cl1	0.35534 (12)	0.10693 (14)	0.18122 (13)	0.0926 (8)	
Cl2	0.51265 (14)	0.18119 (11)	0.21396 (11)	0.0774 (6)	
Cl3	0.51834 (11)	0.03822 (10)	0.15306 (9)	0.0622 (5)	
C1S	0.4624 (4)	0.1240 (4)	0.1603 (3)	0.0525 (16)	
H1SA	0.4627	0.1504	0.1207	0.063*	

### Bis(tetra-n-butylammonium) bis(1,1,1,4,4,4-hexafluorobut-2-ene-2,3-dithiolato)oxalatomolybdate(IV)–chloroform–oxalic acid (1/1/1), (k10131) Atomic displacement parameters (Å^2^)

**Table:**

	*U*^11^	*U*^22^	*U*^33^	*U*^12^	*U*^13^	*U*^23^
Mo1	0.0273 (2)	0.0249 (2)	0.0253 (2)	0.00135 (19)	0.00003 (19)	−0.0008 (2)
S1	0.0311 (7)	0.0373 (8)	0.0287 (8)	−0.0002 (6)	0.0041 (6)	−0.0022 (7)
S2	0.0380 (8)	0.0321 (7)	0.0308 (8)	−0.0003 (6)	0.0001 (6)	−0.0057 (6)
S3	0.0365 (8)	0.0338 (8)	0.0315 (9)	−0.0040 (6)	0.0015 (6)	0.0032 (6)
S4	0.0373 (7)	0.0339 (8)	0.0254 (8)	−0.0024 (6)	−0.0040 (6)	0.0013 (7)
F1	0.049 (2)	0.072 (3)	0.069 (3)	0.0229 (19)	0.020 (2)	0.002 (2)
F2	0.077 (3)	0.076 (3)	0.029 (2)	−0.002 (2)	0.0148 (19)	−0.001 (2)
F3	0.057 (2)	0.065 (3)	0.056 (3)	−0.0097 (19)	0.0256 (19)	−0.007 (2)
F4	0.073 (3)	0.051 (2)	0.049 (3)	0.0162 (19)	0.016 (2)	−0.013 (2)
F5	0.098 (3)	0.058 (2)	0.026 (2)	0.009 (2)	−0.0160 (19)	−0.0038 (19)
F6	0.076 (3)	0.037 (2)	0.040 (2)	−0.0028 (17)	−0.0008 (19)	−0.0134 (17)
F7	0.112 (8)	0.087 (8)	0.075 (7)	−0.073 (6)	−0.024 (6)	0.044 (6)
F8	0.077 (6)	0.026 (5)	0.083 (7)	−0.002 (4)	0.013 (5)	0.024 (5)
F9	0.068 (8)	0.068 (5)	0.063 (6)	0.009 (5)	0.046 (5)	0.010 (4)
F7A	0.068 (6)	0.052 (8)	0.063 (7)	−0.007 (5)	0.019 (5)	0.013 (6)
F8A	0.094 (8)	0.012 (5)	0.077 (8)	−0.008 (5)	0.041 (7)	0.008 (5)
F9A	0.052 (8)	0.072 (7)	0.038 (6)	0.001 (6)	0.029 (5)	0.013 (5)
F10	0.111 (7)	0.113 (9)	0.026 (5)	−0.038 (7)	−0.005 (5)	0.015 (6)
F11	0.089 (7)	0.085 (7)	0.062 (7)	0.051 (6)	−0.043 (6)	−0.035 (6)
F12	0.102 (8)	0.036 (5)	0.045 (6)	−0.014 (5)	−0.035 (5)	0.004 (4)
F10A	0.139 (9)	0.074 (7)	0.038 (6)	0.027 (7)	−0.028 (6)	−0.021 (6)
F11A	0.077 (6)	0.058 (6)	0.043 (6)	0.014 (5)	−0.023 (5)	0.019 (5)
F12A	0.075 (6)	0.127 (10)	0.049 (6)	−0.039 (7)	−0.027 (5)	0.033 (7)
O1	0.0303 (18)	0.0242 (19)	0.039 (2)	0.0030 (14)	−0.0042 (17)	−0.0043 (18)
O2	0.0271 (17)	0.0253 (16)	0.037 (2)	0.0000 (15)	−0.0035 (16)	−0.0018 (19)
O3	0.0251 (18)	0.033 (2)	0.043 (2)	0.0038 (15)	−0.0028 (15)	0.0037 (19)
O4	0.037 (2)	0.025 (2)	0.074 (3)	0.0002 (14)	−0.007 (2)	0.003 (2)
C1	0.040 (3)	0.039 (3)	0.022 (3)	0.010 (3)	0.000 (2)	0.002 (3)
C2	0.033 (3)	0.031 (3)	0.029 (3)	0.009 (2)	−0.004 (2)	0.001 (3)
C3	0.046 (4)	0.059 (5)	0.039 (4)	0.008 (3)	0.015 (3)	−0.002 (3)
C4	0.056 (4)	0.040 (4)	0.031 (4)	0.008 (3)	0.004 (3)	−0.003 (3)
C5	0.042 (3)	0.034 (3)	0.027 (3)	0.006 (3)	0.005 (3)	0.000 (3)
C6	0.039 (3)	0.035 (3)	0.026 (3)	0.008 (3)	−0.001 (2)	−0.003 (3)
C7	0.066 (4)	0.051 (4)	0.039 (4)	−0.004 (3)	0.007 (3)	0.009 (3)
C8	0.059 (4)	0.046 (4)	0.031 (4)	0.002 (3)	−0.002 (3)	0.005 (3)
C9	0.029 (3)	0.029 (3)	0.026 (3)	0.002 (2)	0.003 (2)	0.003 (3)
C10	0.032 (3)	0.028 (3)	0.031 (3)	0.002 (2)	0.005 (2)	−0.001 (2)
O5	0.053 (3)	0.037 (3)	0.131 (5)	0.001 (2)	−0.015 (3)	0.014 (3)
O6	0.042 (2)	0.036 (2)	0.068 (3)	−0.0088 (18)	−0.013 (2)	0.006 (2)
O7	0.067 (3)	0.082 (4)	0.049 (3)	−0.024 (3)	−0.017 (3)	0.006 (3)
O8	0.054 (3)	0.040 (2)	0.055 (3)	−0.019 (2)	−0.013 (2)	0.016 (3)
C11	0.040 (3)	0.042 (4)	0.041 (4)	−0.004 (3)	−0.005 (3)	0.012 (3)
C12	0.036 (3)	0.036 (3)	0.045 (4)	0.001 (2)	−0.004 (3)	0.005 (3)
N1	0.039 (3)	0.037 (3)	0.023 (3)	0.003 (2)	−0.003 (2)	0.004 (2)
C13	0.037 (3)	0.048 (4)	0.036 (4)	0.001 (3)	−0.004 (3)	0.000 (3)
C14	0.042 (3)	0.050 (4)	0.043 (4)	−0.005 (3)	−0.003 (3)	0.000 (3)
C15	0.060 (4)	0.066 (4)	0.057 (5)	−0.023 (4)	−0.010 (4)	0.004 (4)
C16	0.077 (5)	0.081 (6)	0.068 (6)	−0.031 (4)	−0.001 (4)	−0.006 (5)
C17	0.045 (3)	0.037 (3)	0.030 (4)	0.000 (3)	0.005 (3)	−0.001 (3)
C18	0.048 (4)	0.040 (4)	0.042 (4)	0.001 (3)	0.001 (3)	0.007 (3)
C19	0.048 (4)	0.048 (4)	0.049 (4)	−0.004 (3)	0.016 (3)	0.005 (3)
C20	0.049 (5)	0.101 (6)	0.068 (6)	0.007 (4)	0.008 (4)	0.005 (5)
C21	0.047 (3)	0.040 (3)	0.030 (4)	0.004 (3)	−0.002 (3)	0.000 (3)
C22	0.053 (4)	0.048 (4)	0.036 (4)	0.003 (3)	0.001 (3)	0.004 (3)
C23	0.086 (5)	0.085 (6)	0.041 (5)	0.037 (5)	0.017 (4)	0.014 (4)
C24	0.107 (7)	0.123 (8)	0.040 (5)	0.051 (6)	0.012 (5)	0.002 (5)
C25	0.043 (3)	0.036 (3)	0.035 (4)	0.004 (3)	−0.006 (3)	0.000 (3)
C26	0.042 (3)	0.041 (4)	0.035 (4)	0.003 (3)	−0.003 (3)	0.003 (3)
C27	0.069 (4)	0.037 (4)	0.049 (5)	−0.004 (3)	0.002 (3)	0.000 (3)
C28	0.084 (5)	0.041 (4)	0.059 (5)	0.006 (3)	0.003 (4)	0.009 (4)
N2	0.034 (4)	0.044 (4)	0.042 (4)	0.014 (3)	0.009 (5)	0.005 (4)
C29	0.033 (4)	0.059 (6)	0.049 (5)	0.013 (4)	0.007 (4)	0.000 (6)
C30	0.038 (4)	0.070 (6)	0.069 (5)	0.000 (5)	0.008 (4)	0.003 (6)
C31	0.044 (5)	0.085 (7)	0.075 (7)	0.005 (5)	0.012 (5)	0.017 (6)
C32	0.059 (6)	0.129 (11)	0.090 (10)	−0.025 (7)	0.021 (7)	0.011 (8)
C33	0.055 (5)	0.048 (5)	0.052 (5)	0.018 (4)	0.014 (5)	0.001 (4)
C34	0.065 (6)	0.050 (5)	0.065 (6)	0.004 (5)	0.010 (5)	0.001 (5)
C35	0.051 (5)	0.070 (6)	0.081 (7)	0.003 (5)	0.005 (5)	−0.011 (6)
C36	0.060 (7)	0.078 (9)	0.087 (8)	−0.013 (5)	0.017 (6)	0.005 (7)
N2B	0.039 (6)	0.045 (6)	0.046 (6)	0.015 (6)	0.008 (6)	0.004 (6)
C29B	0.033 (6)	0.058 (7)	0.050 (6)	0.008 (6)	0.009 (6)	0.004 (7)
C30B	0.039 (6)	0.071 (8)	0.067 (6)	−0.002 (6)	0.003 (6)	0.009 (7)
C31B	0.056 (7)	0.090 (9)	0.074 (8)	−0.006 (8)	0.011 (7)	0.005 (8)
C32B	0.090 (13)	0.156 (16)	0.099 (15)	0.004 (14)	0.024 (13)	0.010 (14)
C33B	0.041 (7)	0.041 (6)	0.050 (6)	0.014 (6)	0.010 (7)	0.004 (6)
C34B	0.043 (7)	0.043 (6)	0.060 (7)	0.014 (6)	0.011 (7)	0.000 (6)
C35B	0.047 (6)	0.047 (7)	0.064 (7)	0.003 (7)	0.009 (6)	0.004 (7)
C36B	0.060 (7)	0.078 (9)	0.087 (8)	−0.013 (5)	0.017 (6)	0.005 (7)
C37	0.034 (3)	0.041 (4)	0.042 (4)	0.006 (3)	0.007 (3)	0.001 (3)
C38	0.050 (4)	0.054 (4)	0.051 (5)	0.008 (3)	0.003 (3)	−0.013 (4)
C39	0.044 (4)	0.077 (5)	0.055 (5)	0.004 (3)	−0.002 (3)	−0.017 (4)
C40	0.060 (5)	0.098 (6)	0.056 (6)	0.004 (4)	−0.013 (4)	−0.014 (5)
C41	0.042 (3)	0.042 (3)	0.038 (4)	0.015 (2)	0.007 (3)	−0.003 (3)
C42	0.051 (4)	0.071 (5)	0.052 (5)	0.020 (3)	0.005 (3)	0.008 (4)
C43	0.066 (4)	0.055 (4)	0.042 (4)	0.014 (3)	−0.006 (3)	0.000 (4)
C44	0.086 (5)	0.057 (5)	0.043 (5)	0.011 (4)	−0.005 (4)	0.000 (4)
Cl1	0.0468 (11)	0.1084 (18)	0.123 (2)	−0.0053 (10)	0.0036 (11)	−0.0458 (17)
Cl2	0.0895 (15)	0.0661 (13)	0.0767 (16)	−0.0212 (10)	−0.0209 (11)	−0.0111 (11)
Cl3	0.0676 (11)	0.0700 (12)	0.0491 (11)	0.0132 (9)	0.0020 (8)	−0.0076 (9)
C1S	0.047 (3)	0.065 (4)	0.045 (4)	0.005 (3)	−0.005 (4)	0.000 (3)

### Bis(tetra-n-butylammonium) bis(1,1,1,4,4,4-hexafluorobut-2-ene-2,3-dithiolato)oxalatomolybdate(IV)–chloroform–oxalic acid (1/1/1), (k10131) Geometric parameters (Å, º)

**Table:**

Mo1—O1	2.104 (3)	C26—H26B	0.9900
Mo1—O2	2.135 (3)	C27—C28	1.501 (8)
Mo1—S1	2.3265 (14)	C27—H27A	0.9900
Mo1—S2	2.3309 (15)	C27—H27B	0.9900
Mo1—S4	2.3320 (14)	C28—H28A	0.9800
Mo1—S3	2.3390 (15)	C28—H28B	0.9800
S1—C1	1.750 (6)	C28—H28C	0.9800
S2—C2	1.727 (6)	N2—C37	1.49 (3)
S3—C5	1.734 (6)	N2—C29	1.521 (7)
S4—C6	1.746 (6)	N2—C41	1.53 (3)
F1—C3	1.330 (7)	N2—C33	1.535 (7)
F2—C3	1.342 (8)	C29—C30	1.501 (8)
F3—C3	1.347 (7)	C29—H29A	0.9900
F4—C4	1.350 (7)	C29—H29B	0.9900
F5—C4	1.346 (7)	C30—C31	1.505 (8)
F6—C4	1.329 (7)	C30—H30A	0.9900
F7—C7	1.342 (6)	C30—H30B	0.9900
F8—C7	1.355 (6)	C31—C32	1.524 (9)
F9—C7	1.330 (6)	C31—H31A	0.9900
F7A—C7	1.349 (6)	C31—H31B	0.9900
F8A—C7	1.326 (6)	C32—H32A	0.9800
F9A—C7	1.349 (6)	C32—H32B	0.9800
F10—C8	1.306 (11)	C32—H32C	0.9800
F11—C8	1.332 (11)	C33—C34	1.504 (8)
F12—C8	1.318 (10)	C33—H33A	0.9900
F10A—C8	1.326 (11)	C33—H33B	0.9900
F11A—C8	1.325 (10)	C34—C35	1.499 (8)
F12A—C8	1.304 (11)	C34—H34A	0.9900
O1—C9	1.276 (6)	C34—H34B	0.9900
O2—C10	1.275 (6)	C35—C36	1.504 (9)
O3—C9	1.229 (6)	C35—H35A	0.9900
O4—C10	1.235 (6)	C35—H35B	0.9900
C1—C2	1.349 (8)	C36—H36A	0.9800
C1—C3	1.500 (8)	C36—H36B	0.9800
C2—C4	1.502 (8)	C36—H36C	0.9800
C5—C6	1.353 (8)	N2B—C41	1.51 (7)
C5—C7	1.502 (8)	N2B—C29B	1.519 (7)
C6—C8	1.502 (8)	N2B—C33B	1.533 (8)
C9—C10	1.528 (7)	N2B—C37	1.59 (6)
O5—C11	1.185 (7)	C29B—C30B	1.502 (9)
O6—C11	1.310 (7)	C29B—H29C	0.9900
O6—H6O	0.88 (7)	C29B—H29D	0.9900
O7—C12	1.198 (7)	C30B—C31B	1.507 (9)
O8—C12	1.308 (8)	C30B—H30C	0.9900
O8—H8O	0.85 (8)	C30B—H30D	0.9900
C11—C12	1.520 (8)	C31B—C32B	1.510 (10)
N1—C17	1.513 (7)	C31B—H31C	0.9900
N1—C13	1.519 (6)	C31B—H31D	0.9900
N1—C21	1.524 (8)	C32B—H32D	0.9800
N1—C25	1.527 (6)	C32B—H32E	0.9800
C13—C14	1.503 (7)	C32B—H32F	0.9800
C13—H13A	0.9900	C33B—C34B	1.502 (9)
C13—H13B	0.9900	C33B—H33C	0.9900
C14—C15	1.504 (7)	C33B—H33D	0.9900
C14—H14A	0.9900	C34B—C35B	1.503 (9)
C14—H14B	0.9900	C34B—H34C	0.9900
C15—C16	1.500 (8)	C34B—H34D	0.9900
C15—H15A	0.9900	C35B—C36B	1.507 (10)
C15—H15B	0.9900	C35B—H35C	0.9900
C16—H16A	0.9800	C35B—H35D	0.9900
C16—H16B	0.9800	C36B—H36D	0.9800
C16—H16C	0.9800	C36B—H36E	0.9800
C17—C18	1.506 (8)	C36B—H36F	0.9800
C17—H17A	0.9900	C37—C38	1.508 (9)
C17—H17B	0.9900	C37—H37A	0.9900
C18—C19	1.506 (8)	C37—H37B	0.9900
C18—H18A	0.9900	C38—C39	1.504 (9)
C18—H18B	0.9900	C38—H38A	0.9900
C19—C20	1.511 (9)	C38—H38B	0.9900
C19—H19A	0.9900	C39—C40	1.522 (10)
C19—H19B	0.9900	C39—H39A	0.9900
C20—H20A	0.9800	C39—H39B	0.9900
C20—H20B	0.9800	C40—H40A	0.9800
C20—H20C	0.9800	C40—H40B	0.9800
C21—C22	1.511 (8)	C40—H40C	0.9800
C21—H21A	0.9900	C41—C42	1.520 (9)
C21—H21B	0.9900	C41—H41A	0.9900
C22—C23	1.499 (10)	C41—H41B	0.9900
C22—H22A	0.9900	C42—C43	1.519 (9)
C22—H22B	0.9900	C42—H42A	0.9900
C23—C24	1.507 (10)	C42—H42B	0.9900
C23—H23A	0.9900	C43—C44	1.515 (9)
C23—H23B	0.9900	C43—H43A	0.9900
C24—H24A	0.9800	C43—H43B	0.9900
C24—H24B	0.9800	C44—H44A	0.9800
C24—H24C	0.9800	C44—H44B	0.9800
C25—C26	1.506 (7)	C44—H44C	0.9800
C25—H25A	0.9900	Cl1—C1S	1.739 (7)
C25—H25B	0.9900	Cl2—C1S	1.754 (7)
C26—C27	1.507 (7)	Cl3—C1S	1.767 (7)
C26—H26A	0.9900	C1S—H1SA	1.0000
			
O1—Mo1—O2	73.83 (12)	C26—C27—H27A	108.7
O1—Mo1—S1	127.88 (12)	C28—C27—H27B	108.7
O2—Mo1—S1	84.04 (9)	C26—C27—H27B	108.7
O1—Mo1—S2	83.86 (10)	H27A—C27—H27B	107.6
O2—Mo1—S2	137.77 (10)	C27—C28—H28A	109.5
S1—Mo1—S2	82.12 (5)	C27—C28—H28B	109.5
O1—Mo1—S4	140.08 (11)	H28A—C28—H28B	109.5
O2—Mo1—S4	86.48 (10)	C27—C28—H28C	109.5
S1—Mo1—S4	82.51 (5)	H28A—C28—H28C	109.5
S2—Mo1—S4	130.47 (5)	H28B—C28—H28C	109.5
O1—Mo1—S3	84.26 (11)	C37—N2—C29	110.0 (16)
O2—Mo1—S3	128.89 (10)	C37—N2—C41	107.8 (7)
S1—Mo1—S3	142.11 (5)	C29—N2—C41	110.9 (16)
S2—Mo1—S3	82.37 (5)	C37—N2—C33	108.0 (16)
S4—Mo1—S3	81.74 (5)	C29—N2—C33	107.8 (6)
C1—S1—Mo1	109.50 (19)	C41—N2—C33	112.3 (14)
C2—S2—Mo1	109.2 (2)	C30—C29—N2	115.1 (6)
C5—S3—Mo1	109.61 (19)	C30—C29—H29A	108.5
C6—S4—Mo1	109.9 (2)	N2—C29—H29A	108.5
C9—O1—Mo1	120.6 (3)	C30—C29—H29B	108.5
C10—O2—Mo1	118.5 (3)	N2—C29—H29B	108.5
C2—C1—C3	125.8 (5)	H29A—C29—H29B	107.5
C2—C1—S1	118.4 (4)	C29—C30—C31	114.4 (7)
C3—C1—S1	115.8 (4)	C29—C30—H30A	108.7
C1—C2—C4	125.0 (5)	C31—C30—H30A	108.7
C1—C2—S2	120.6 (4)	C29—C30—H30B	108.7
C4—C2—S2	114.3 (4)	C31—C30—H30B	108.7
F1—C3—F2	107.2 (5)	H30A—C30—H30B	107.6
F1—C3—F3	105.8 (5)	C30—C31—C32	112.3 (8)
F2—C3—F3	106.0 (5)	C30—C31—H31A	109.1
F1—C3—C1	112.7 (5)	C32—C31—H31A	109.1
F2—C3—C1	112.4 (5)	C30—C31—H31B	109.1
F3—C3—C1	112.3 (5)	C32—C31—H31B	109.1
F6—C4—F5	106.0 (5)	H31A—C31—H31B	107.9
F6—C4—F4	105.4 (5)	C31—C32—H32A	109.5
F5—C4—F4	105.4 (5)	C31—C32—H32B	109.5
F6—C4—C2	113.4 (5)	H32A—C32—H32B	109.5
F5—C4—C2	112.9 (5)	C31—C32—H32C	109.5
F4—C4—C2	112.9 (5)	H32A—C32—H32C	109.5
C6—C5—C7	124.6 (5)	H32B—C32—H32C	109.5
C6—C5—S3	119.8 (4)	C34—C33—N2	116.2 (6)
C7—C5—S3	115.6 (4)	C34—C33—H33A	108.2
C5—C6—C8	126.6 (5)	N2—C33—H33A	108.2
C5—C6—S4	118.7 (4)	C34—C33—H33B	108.2
C8—C6—S4	114.7 (4)	N2—C33—H33B	108.2
F9—C7—F7	106.8 (5)	H33A—C33—H33B	107.4
F8A—C7—F9A	106.3 (6)	C35—C34—C33	113.8 (7)
F8A—C7—F7A	106.7 (5)	C35—C34—H34A	108.8
F9A—C7—F7A	103.9 (5)	C33—C34—H34A	108.8
F9—C7—F8	105.3 (5)	C35—C34—H34B	108.8
F7—C7—F8	103.8 (5)	C33—C34—H34B	108.8
F8A—C7—C5	118.5 (7)	H34A—C34—H34B	107.7
F9—C7—C5	117.2 (7)	C34—C35—C36	112.7 (8)
F7—C7—C5	112.9 (6)	C34—C35—H35A	109.1
F9A—C7—C5	110.9 (8)	C36—C35—H35A	109.1
F7A—C7—C5	109.6 (7)	C34—C35—H35B	109.1
F8—C7—C5	109.6 (6)	C36—C35—H35B	109.1
F10—C8—F12	105.3 (9)	H35A—C35—H35B	107.8
F12A—C8—F11A	106.7 (9)	C35—C36—H36A	109.5
F12A—C8—F10A	107.0 (10)	C35—C36—H36B	109.5
F11A—C8—F10A	104.8 (9)	H36A—C36—H36B	109.5
F10—C8—F11	107.9 (10)	C35—C36—H36C	109.5
F12—C8—F11	104.5 (9)	H36A—C36—H36C	109.5
F12A—C8—C6	112.4 (7)	H36B—C36—H36C	109.5
F10—C8—C6	113.6 (7)	C41—N2B—C29B	111 (4)
F12—C8—C6	113.4 (6)	C41—N2B—C33B	110 (3)
F11A—C8—C6	112.8 (6)	C29B—N2B—C33B	107.5 (8)
F10A—C8—C6	112.5 (6)	C41—N2B—C37	103.9 (11)
F11—C8—C6	111.5 (6)	C29B—N2B—C37	111 (3)
O3—C9—O1	124.9 (5)	C33B—N2B—C37	114 (4)
O3—C9—C10	122.4 (4)	C30B—C29B—N2B	116.3 (9)
O1—C9—C10	112.6 (4)	C30B—C29B—H29C	108.2
O4—C10—O2	125.8 (5)	N2B—C29B—H29C	108.2
O4—C10—C9	119.8 (4)	C30B—C29B—H29D	108.2
O2—C10—C9	114.4 (4)	N2B—C29B—H29D	108.2
C11—O6—H6O	120 (4)	H29C—C29B—H29D	107.4
C12—O8—H8O	115 (6)	C29B—C30B—C31B	112.3 (10)
O5—C11—O6	127.2 (6)	C29B—C30B—H30C	109.1
O5—C11—C12	121.6 (5)	C31B—C30B—H30C	109.1
O6—C11—C12	111.2 (5)	C29B—C30B—H30D	109.1
O7—C12—O8	125.4 (6)	C31B—C30B—H30D	109.1
O7—C12—C11	122.9 (6)	H30C—C30B—H30D	107.9
O8—C12—C11	111.7 (6)	C30B—C31B—C32B	113.7 (10)
C17—N1—C13	109.2 (4)	C30B—C31B—H31C	108.8
C17—N1—C21	110.0 (4)	C32B—C31B—H31C	108.8
C13—N1—C21	110.8 (4)	C30B—C31B—H31D	108.8
C17—N1—C25	110.8 (4)	C32B—C31B—H31D	108.8
C13—N1—C25	108.5 (4)	H31C—C31B—H31D	107.7
C21—N1—C25	107.6 (4)	C31B—C32B—H32D	109.5
C14—C13—N1	116.3 (4)	C31B—C32B—H32E	109.5
C14—C13—H13A	108.2	H32D—C32B—H32E	109.5
N1—C13—H13A	108.2	C31B—C32B—H32F	109.5
C14—C13—H13B	108.2	H32D—C32B—H32F	109.5
N1—C13—H13B	108.2	H32E—C32B—H32F	109.5
H13A—C13—H13B	107.4	C34B—C33B—N2B	117.3 (9)
C13—C14—C15	111.8 (5)	C34B—C33B—H33C	108.0
C13—C14—H14A	109.3	N2B—C33B—H33C	108.0
C15—C14—H14A	109.3	C34B—C33B—H33D	108.0
C13—C14—H14B	109.3	N2B—C33B—H33D	108.0
C15—C14—H14B	109.3	H33C—C33B—H33D	107.2
H14A—C14—H14B	107.9	C33B—C34B—C35B	114.2 (10)
C16—C15—C14	115.6 (6)	C33B—C34B—H34C	108.7
C16—C15—H15A	108.4	C35B—C34B—H34C	108.7
C14—C15—H15A	108.4	C33B—C34B—H34D	108.7
C16—C15—H15B	108.4	C35B—C34B—H34D	108.7
C14—C15—H15B	108.4	H34C—C34B—H34D	107.6
H15A—C15—H15B	107.5	C34B—C35B—C36B	112.6 (10)
C15—C16—H16A	109.5	C34B—C35B—H35C	109.1
C15—C16—H16B	109.5	C36B—C35B—H35C	109.1
H16A—C16—H16B	109.5	C34B—C35B—H35D	109.1
C15—C16—H16C	109.5	C36B—C35B—H35D	109.1
H16A—C16—H16C	109.5	H35C—C35B—H35D	107.8
H16B—C16—H16C	109.5	C35B—C36B—H36D	109.5
C18—C17—N1	117.7 (5)	C35B—C36B—H36E	109.5
C18—C17—H17A	107.9	H36D—C36B—H36E	109.5
N1—C17—H17A	107.9	C35B—C36B—H36F	109.5
C18—C17—H17B	107.9	H36D—C36B—H36F	109.5
N1—C17—H17B	107.9	H36E—C36B—H36F	109.5
H17A—C17—H17B	107.2	N2—C37—C38	115.8 (10)
C17—C18—C19	110.1 (5)	C38—C37—N2B	115 (2)
C17—C18—H18A	109.6	N2—C37—H37A	108.3
C19—C18—H18A	109.6	C38—C37—H37A	108.3
C17—C18—H18B	109.6	N2—C37—H37B	108.3
C19—C18—H18B	109.6	C38—C37—H37B	108.3
H18A—C18—H18B	108.2	H37A—C37—H37B	107.4
C18—C19—C20	114.1 (6)	C39—C38—C37	112.0 (5)
C18—C19—H19A	108.7	C39—C38—H38A	109.2
C20—C19—H19A	108.7	C37—C38—H38A	109.2
C18—C19—H19B	108.7	C39—C38—H38B	109.2
C20—C19—H19B	108.7	C37—C38—H38B	109.2
H19A—C19—H19B	107.6	H38A—C38—H38B	107.9
C19—C20—H20A	109.5	C38—C39—C40	113.4 (6)
C19—C20—H20B	109.5	C38—C39—H39A	108.9
H20A—C20—H20B	109.5	C40—C39—H39A	108.9
C19—C20—H20C	109.5	C38—C39—H39B	108.9
H20A—C20—H20C	109.5	C40—C39—H39B	108.9
H20B—C20—H20C	109.5	H39A—C39—H39B	107.7
C22—C21—N1	116.4 (5)	C39—C40—H40A	109.5
C22—C21—H21A	108.2	C39—C40—H40B	109.5
N1—C21—H21A	108.2	H40A—C40—H40B	109.5
C22—C21—H21B	108.2	C39—C40—H40C	109.5
N1—C21—H21B	108.2	H40A—C40—H40C	109.5
H21A—C21—H21B	107.3	H40B—C40—H40C	109.5
C23—C22—C21	111.9 (5)	N2B—C41—C42	114.0 (19)
C23—C22—H22A	109.2	C42—C41—N2	117.8 (9)
C21—C22—H22A	109.2	C42—C41—H41A	107.9
C23—C22—H22B	109.2	N2—C41—H41A	107.9
C21—C22—H22B	109.2	C42—C41—H41B	107.9
H22A—C22—H22B	107.9	N2—C41—H41B	107.9
C22—C23—C24	113.5 (6)	H41A—C41—H41B	107.2
C22—C23—H23A	108.9	C43—C42—C41	111.5 (5)
C24—C23—H23A	108.9	C43—C42—H42A	109.3
C22—C23—H23B	108.9	C41—C42—H42A	109.3
C24—C23—H23B	108.9	C43—C42—H42B	109.3
H23A—C23—H23B	107.7	C41—C42—H42B	109.3
C23—C24—H24A	109.5	H42A—C42—H42B	108.0
C23—C24—H24B	109.5	C44—C43—C42	115.5 (6)
H24A—C24—H24B	109.5	C44—C43—H43A	108.4
C23—C24—H24C	109.5	C42—C43—H43A	108.4
H24A—C24—H24C	109.5	C44—C43—H43B	108.4
H24B—C24—H24C	109.5	C42—C43—H43B	108.4
C26—C25—N1	118.0 (4)	H43A—C43—H43B	107.5
C26—C25—H25A	107.8	C43—C44—H44A	109.5
N1—C25—H25A	107.8	C43—C44—H44B	109.5
C26—C25—H25B	107.8	H44A—C44—H44B	109.5
N1—C25—H25B	107.8	C43—C44—H44C	109.5
H25A—C25—H25B	107.1	H44A—C44—H44C	109.5
C25—C26—C27	110.4 (5)	H44B—C44—H44C	109.5
C25—C26—H26A	109.6	Cl1—C1S—Cl2	109.7 (4)
C27—C26—H26A	109.6	Cl1—C1S—Cl3	109.5 (4)
C25—C26—H26B	109.6	Cl2—C1S—Cl3	110.6 (4)
C27—C26—H26B	109.6	Cl1—C1S—H1SA	109.0
H26A—C26—H26B	108.1	Cl2—C1S—H1SA	109.0
C28—C27—C26	114.1 (5)	Cl3—C1S—H1SA	109.0
C28—C27—H27A	108.7		
			
Mo1—S1—C1—C2	−4.8 (5)	O6—C11—C12—O7	−114.9 (7)
Mo1—S1—C1—C3	177.8 (4)	O5—C11—C12—O8	−113.7 (7)
C3—C1—C2—C4	−5.6 (9)	O6—C11—C12—O8	67.0 (7)
S1—C1—C2—C4	177.2 (4)	C17—N1—C13—C14	68.0 (6)
C3—C1—C2—S2	178.7 (5)	C21—N1—C13—C14	−53.2 (6)
S1—C1—C2—S2	1.5 (6)	C25—N1—C13—C14	−171.2 (5)
Mo1—S2—C2—C1	2.5 (5)	N1—C13—C14—C15	−172.6 (5)
Mo1—S2—C2—C4	−173.7 (4)	C13—C14—C15—C16	−175.6 (6)
C2—C1—C3—F1	−74.2 (8)	C13—N1—C17—C18	176.3 (5)
S1—C1—C3—F1	103.1 (6)	C21—N1—C17—C18	−62.0 (6)
C2—C1—C3—F2	47.1 (8)	C25—N1—C17—C18	56.9 (6)
S1—C1—C3—F2	−135.6 (5)	N1—C17—C18—C19	−177.3 (5)
C2—C1—C3—F3	166.5 (6)	C17—C18—C19—C20	−176.4 (6)
S1—C1—C3—F3	−16.2 (7)	C17—N1—C21—C22	−177.0 (5)
C1—C2—C4—F6	160.3 (5)	C13—N1—C21—C22	−56.2 (6)
S2—C2—C4—F6	−23.8 (6)	C25—N1—C21—C22	62.3 (6)
C1—C2—C4—F5	−79.1 (7)	N1—C21—C22—C23	−170.7 (6)
S2—C2—C4—F5	96.8 (5)	C21—C22—C23—C24	169.7 (7)
C1—C2—C4—F4	40.4 (8)	C17—N1—C25—C26	51.8 (6)
S2—C2—C4—F4	−143.7 (4)	C13—N1—C25—C26	−68.0 (6)
Mo1—S3—C5—C6	−4.3 (5)	C21—N1—C25—C26	172.1 (5)
Mo1—S3—C5—C7	177.7 (3)	N1—C25—C26—C27	179.2 (5)
C7—C5—C6—C8	2.7 (9)	C25—C26—C27—C28	−177.4 (6)
S3—C5—C6—C8	−175.0 (5)	C37—N2—C29—C30	−60 (2)
C7—C5—C6—S4	−179.6 (4)	C41—N2—C29—C30	58.8 (19)
S3—C5—C6—S4	2.6 (6)	C33—N2—C29—C30	−177.9 (17)
Mo1—S4—C6—C5	0.4 (5)	N2—C29—C30—C31	167.2 (18)
Mo1—S4—C6—C8	178.3 (4)	C29—C30—C31—C32	174.7 (13)
C6—C5—C7—F8A	−83.8 (11)	C37—N2—C33—C34	69.2 (18)
S3—C5—C7—F8A	94.1 (10)	C29—N2—C33—C34	−172.0 (16)
C6—C5—C7—F9	66.9 (10)	C41—N2—C33—C34	−49.5 (18)
S3—C5—C7—F9	−115.3 (8)	N2—C33—C34—C35	−149.6 (17)
C6—C5—C7—F7	−168.3 (10)	C33—C34—C35—C36	−177.2 (11)
S3—C5—C7—F7	9.6 (10)	C41—N2B—C29B—C30B	60 (4)
C6—C5—C7—F9A	39.4 (10)	C33B—N2B—C29B—C30B	−180 (4)
S3—C5—C7—F9A	−142.7 (8)	C37—N2B—C29B—C30B	−55 (5)
C6—C5—C7—F7A	153.6 (9)	N2B—C29B—C30B—C31B	−174 (4)
S3—C5—C7—F7A	−28.6 (8)	C29B—C30B—C31B—C32B	−92 (3)
C6—C5—C7—F8	−53.0 (9)	C41—N2B—C33B—C34B	−47 (4)
S3—C5—C7—F8	124.8 (8)	C29B—N2B—C33B—C34B	−168 (3)
C5—C6—C8—F12A	154.0 (10)	C37—N2B—C33B—C34B	69 (4)
S4—C6—C8—F12A	−23.8 (11)	N2B—C33B—C34B—C35B	−81 (3)
C5—C6—C8—F10	−28.9 (12)	C33B—C34B—C35B—C36B	−170 (2)
S4—C6—C8—F10	153.4 (9)	C29—N2—C37—C38	−50.0 (13)
C5—C6—C8—F12	−149.1 (9)	C41—N2—C37—C38	−171.0 (5)
S4—C6—C8—F12	33.2 (10)	C33—N2—C37—C38	67.4 (11)
C5—C6—C8—F11A	33.3 (10)	C41—N2B—C37—C38	−176.8 (7)
S4—C6—C8—F11A	−144.5 (7)	C29B—N2B—C37—C38	−57 (3)
C5—C6—C8—F10A	−85.1 (11)	C33B—N2B—C37—C38	64 (2)
S4—C6—C8—F10A	97.1 (9)	N2—C37—C38—C39	−171.5 (6)
C5—C6—C8—F11	93.3 (10)	N2B—C37—C38—C39	−178.8 (8)
S4—C6—C8—F11	−84.4 (10)	C37—C38—C39—C40	−176.9 (6)
Mo1—O1—C9—O3	−179.3 (4)	C29B—N2B—C41—C42	63 (2)
Mo1—O1—C9—C10	1.6 (6)	C33B—N2B—C41—C42	−55 (3)
Mo1—O2—C10—O4	−177.2 (4)	C37—N2B—C41—C42	−177.7 (6)
Mo1—O2—C10—C9	2.9 (6)	C37—N2—C41—C42	176.8 (6)
O3—C9—C10—O4	−2.0 (8)	C29—N2—C41—C42	56.3 (12)
O1—C9—C10—O4	177.1 (5)	C33—N2—C41—C42	−64.4 (12)
O3—C9—C10—O2	178.0 (5)	N2B—C41—C42—C43	170.5 (8)
O1—C9—C10—O2	−2.9 (7)	N2—C41—C42—C43	163.0 (6)
O5—C11—C12—O7	64.5 (10)	C41—C42—C43—C44	55.6 (9)

### Bis(tetra-n-butylammonium) bis(1,1,1,4,4,4-hexafluorobut-2-ene-2,3-dithiolato)oxalatomolybdate(IV)–chloroform–oxalic acid (1/1/1), (k10131) Hydrogen-bond geometry (Å, º)

**Table:**

*D*—H···*A*	*D*—H	H···*A*	*D*···*A*	*D*—H···*A*
O6—H6*O*···O3^i^	0.88 (7)	1.76 (7)	2.633 (5)	170 (7)
O8—H8*O*···O4	0.85 (8)	1.75 (8)	2.587 (5)	174 (9)

Symmetry code: (i) *x*+1/2, −*y*+3/2, −*z*+1.

### Bis(tetra-n-butylammonium) µ-oxalato-bis[bis(1,1,1,4,4,4-hexafluorobut-2-ene-2,3-dithiolato)molybdate(IV)] (k10171_sq) Crystal data

**Table:**

(C_16_H_36_N)[Mo_2_(C_4_F_6_S_2_)_4_(C_2_O_4_)]	*F*(000) = 1692
*M**_r_* = 1669.45	*D*_x_ = 1.424 Mg m^−^^3^
Monoclinic, *P*2_1_/*n*	Mo *K*α radiation, λ = 0.71073 Å
*a* = 14.2347 (15) Å	Cell parameters from 18892 reflections
*b* = 19.4940 (19) Å	θ = 2.6–25.7°
*c* = 14.4056 (14) Å	µ = 0.63 mm^−^^1^
β = 103.159 (5)°	*T* = 150 K
*V* = 3892.5 (7) Å^3^	Plate, green
*Z* = 2	0.18 × 0.18 × 0.06 mm

### Bis(tetra-n-butylammonium) µ-oxalato-bis[bis(1,1,1,4,4,4-hexafluorobut-2-ene-2,3-dithiolato)molybdate(IV)] (k10171_sq) Data collection

**Table:**

Nonius KappaCCD diffractometer	7278 independent reflections
Radiation source: fine-focus sealed tube	4243 reflections with *I* > 2σ(*I*)
Detector resolution: 9 pixels mm^-1^	*R*_int_ = 0.066
φ scan and ω scans with κ offsets	θ_max_ = 25.8°, θ_min_ = 2.8°
Absorption correction: multi-scan (SORTAV; Blessing, 1995)	*h* = −17→16
*T*_min_ = 0.720, *T*_max_ = 0.931	*k* = −21→23
18527 measured reflections	*l* = −14→17

### Bis(tetra-n-butylammonium) µ-oxalato-bis[bis(1,1,1,4,4,4-hexafluorobut-2-ene-2,3-dithiolato)molybdate(IV)] (k10171_sq) Refinement

**Table:**

Refinement on *F*^2^	520 restraints
Least-squares matrix: full	Hydrogen site location: inferred from neighbouring sites
*R*[*F*^2^ > 2σ(*F*^2^)] = 0.065	H-atom parameters constrained
*wR*(*F*^2^) = 0.164	*w* = 1/[σ^2^(*F*_o_^2^) + (0.0589*P*)^2^ + 6.741*P*] where *P* = (*F*_o_^2^ + 2*F*_c_^2^)/3
*S* = 1.01	(Δ/σ)_max_ = 0.001
7278 reflections	Δρ_max_ = 0.59 e Å^−^^3^
560 parameters	Δρ_min_ = −0.65 e Å^−^^3^

### Bis(tetra-n-butylammonium) µ-oxalato-bis[bis(1,1,1,4,4,4-hexafluorobut-2-ene-2,3-dithiolato)molybdate(IV)] (k10171_sq) Special details

**Table:**

Geometry. All esds (except the esd in the dihedral angle between two l.s. planes) are estimated using the full covariance matrix. The cell esds are taken into account individually in the estimation of esds in distances, angles and torsion angles; correlations between esds in cell parameters are only used when they are defined by crystal symmetry. An approximate (isotropic) treatment of cell esds is used for estimating esds involving l.s. planes.

### Bis(tetra-n-butylammonium) µ-oxalato-bis[bis(1,1,1,4,4,4-hexafluorobut-2-ene-2,3-dithiolato)molybdate(IV)] (k10171_sq) Fractional atomic coordinates and isotropic or equivalent isotropic displacement parameters (Å^2^)

**Table:**

	*x*	*y*	*z*	*U*_iso_*/*U*_eq_	Occ. (<1)
Mo1	0.87109 (4)	0.08321 (2)	0.36570 (4)	0.05150 (18)	
S1	0.89301 (13)	0.07056 (8)	0.21225 (13)	0.0659 (5)	
S2	0.93704 (12)	0.19051 (8)	0.35112 (13)	0.0678 (5)	
S3	0.77245 (12)	0.14498 (8)	0.44228 (12)	0.0633 (4)	
S4	0.72826 (11)	0.02567 (7)	0.30266 (12)	0.0591 (4)	
F1	0.9664 (5)	0.0777 (3)	0.0486 (4)	0.1268 (19)	
F2	0.9059 (5)	0.1775 (3)	0.0199 (4)	0.139 (2)	
F3	1.0559 (4)	0.1657 (3)	0.0798 (5)	0.141 (2)	
F4	1.1100 (4)	0.2576 (3)	0.2483 (6)	0.203 (4)	
F5	0.9905 (5)	0.3134 (3)	0.2739 (4)	0.132 (2)	
F6	0.9926 (4)	0.2848 (2)	0.1371 (4)	0.1192 (18)	
F7	0.6143 (4)	0.2064 (3)	0.4933 (5)	0.155 (3)	
F8	0.5004 (4)	0.1532 (3)	0.4101 (4)	0.138 (2)	
F9	0.5744 (4)	0.1101 (3)	0.5375 (4)	0.131 (2)	
F10	0.4944 (4)	0.0160 (3)	0.3889 (5)	0.146 (2)	
F11	0.5485 (3)	−0.0406 (2)	0.2910 (4)	0.1217 (19)	
F12	0.4847 (3)	0.0548 (3)	0.2499 (4)	0.130 (2)	
O1	0.9309 (3)	−0.01919 (18)	0.3874 (3)	0.0557 (10)	
O2	1.0310 (3)	−0.08488 (18)	0.4949 (3)	0.0573 (10)	
C1	0.9504 (5)	0.1439 (4)	0.1820 (6)	0.075 (2)	
C2	0.9686 (5)	0.1961 (3)	0.2414 (6)	0.0709 (19)	
C3	0.9702 (7)	0.1418 (5)	0.0815 (7)	0.102 (3)	
C4	1.0177 (6)	0.2622 (4)	0.2255 (8)	0.099 (3)	
C5	0.6576 (5)	0.1110 (3)	0.4153 (5)	0.0640 (17)	
C6	0.6378 (4)	0.0580 (3)	0.3530 (5)	0.0636 (17)	
C7	0.5880 (6)	0.1450 (4)	0.4666 (7)	0.085 (2)	
C8	0.5414 (5)	0.0231 (4)	0.3193 (7)	0.084 (2)	
C9	0.9890 (4)	−0.0297 (3)	0.4657 (4)	0.0516 (15)	
N1	0.6284 (10)	0.2680 (7)	0.1575 (11)	0.0755 (19)	0.589 (6)
C10	0.5205 (11)	0.2799 (9)	0.1500 (13)	0.082 (3)	0.589 (6)
H10A	0.5095	0.3299	0.1530	0.099*	0.589 (6)
H10B	0.4850	0.2639	0.0864	0.099*	0.589 (6)
C11	0.4765 (9)	0.2454 (7)	0.2247 (11)	0.093 (3)	0.589 (6)
H11A	0.5043	0.2657	0.2880	0.111*	0.589 (6)
H11B	0.4927	0.1959	0.2276	0.111*	0.589 (6)
C12	0.3656 (10)	0.2541 (8)	0.2012 (13)	0.103 (4)	0.589 (6)
H12A	0.3423	0.2547	0.2609	0.124*	0.589 (6)
H12B	0.3477	0.2981	0.1677	0.124*	0.589 (6)
C13	0.3202 (13)	0.1967 (10)	0.1402 (14)	0.133 (5)	0.589 (6)
H13A	0.2500	0.2023	0.1254	0.199*	0.589 (6)
H13B	0.3430	0.1966	0.0810	0.199*	0.589 (6)
H13C	0.3376	0.1533	0.1740	0.199*	0.589 (6)
C14	0.6590 (13)	0.3140 (12)	0.085 (2)	0.077 (2)	0.589 (6)
H14A	0.6287	0.3595	0.0881	0.093*	0.589 (6)
H14B	0.6325	0.2948	0.0212	0.093*	0.589 (6)
C15	0.7680 (12)	0.3248 (10)	0.0967 (12)	0.084 (3)	0.589 (6)
H15A	0.7960	0.3428	0.1613	0.101*	0.589 (6)
H15B	0.7989	0.2800	0.0903	0.101*	0.589 (6)
C16	0.7904 (11)	0.3743 (7)	0.0232 (9)	0.085 (3)	0.589 (6)
H16A	0.8606	0.3831	0.0362	0.101*	0.589 (6)
H16B	0.7569	0.4185	0.0263	0.101*	0.589 (6)
C17	0.7553 (12)	0.3416 (8)	−0.0780 (10)	0.106 (4)	0.589 (6)
H17A	0.7692	0.3730	−0.1262	0.159*	0.589 (6)
H17B	0.7890	0.2981	−0.0806	0.159*	0.589 (6)
H17C	0.6856	0.3333	−0.0904	0.159*	0.589 (6)
C18	0.6467 (12)	0.1923 (8)	0.1431 (11)	0.085 (3)	0.589 (6)
H18A	0.6054	0.1647	0.1757	0.102*	0.589 (6)
H18B	0.7147	0.1816	0.1735	0.102*	0.589 (6)
C19	0.6260 (11)	0.1704 (7)	0.0352 (11)	0.094 (3)	0.589 (6)
H19A	0.5568	0.1771	0.0053	0.113*	0.589 (6)
H19B	0.6642	0.1994	0.0009	0.113*	0.589 (6)
C20	0.6534 (11)	0.0941 (7)	0.0276 (13)	0.103 (4)	0.589 (6)
H20A	0.6589	0.0714	0.0901	0.123*	0.589 (6)
H20B	0.7168	0.0910	0.0106	0.123*	0.589 (6)
C21	0.5731 (13)	0.0556 (9)	−0.0519 (14)	0.123 (6)	0.589 (6)
H21A	0.5914	0.0074	−0.0558	0.185*	0.589 (6)
H21B	0.5105	0.0582	−0.0344	0.185*	0.589 (6)
H21C	0.5684	0.0778	−0.1138	0.185*	0.589 (6)
C22	0.6825 (19)	0.2873 (10)	0.2593 (14)	0.082 (3)	0.589 (6)
H22A	0.6636	0.2545	0.3043	0.099*	0.589 (6)
H22B	0.7525	0.2814	0.2640	0.099*	0.589 (6)
C23	0.666 (3)	0.3588 (11)	0.2913 (16)	0.090 (3)	0.589 (6)
H23A	0.5955	0.3663	0.2812	0.108*	0.589 (6)
H23B	0.6905	0.3917	0.2501	0.108*	0.589 (6)
C24	0.7128 (13)	0.3750 (9)	0.3947 (13)	0.103 (3)	0.589 (6)
H24A	0.6894	0.4200	0.4118	0.123*	0.589 (6)
H24B	0.6936	0.3398	0.4364	0.123*	0.589 (6)
C25	0.8214 (11)	0.3767 (8)	0.4118 (12)	0.120 (5)	0.589 (6)
H25A	0.8492	0.3872	0.4789	0.180*	0.589 (6)
H25B	0.8449	0.3319	0.3960	0.180*	0.589 (6)
H25C	0.8407	0.4121	0.3715	0.180*	0.589 (6)
N1A	0.6310 (15)	0.2661 (10)	0.1504 (15)	0.077 (2)	0.411 (6)
C10A	0.5216 (14)	0.2704 (13)	0.1279 (19)	0.082 (3)	0.411 (6)
H10C	0.5033	0.3171	0.1443	0.098*	0.411 (6)
H10D	0.4974	0.2645	0.0583	0.098*	0.411 (6)
C11A	0.4713 (12)	0.2192 (10)	0.1781 (16)	0.090 (3)	0.411 (6)
H11C	0.4948	0.2238	0.2480	0.108*	0.411 (6)
H11D	0.4853	0.1720	0.1598	0.108*	0.411 (6)
C12A	0.3619 (13)	0.2325 (13)	0.1502 (17)	0.095 (4)	0.411 (6)
H12C	0.3491	0.2825	0.1482	0.114*	0.411 (6)
H12D	0.3344	0.2132	0.0862	0.114*	0.411 (6)
C13A	0.3161 (15)	0.1988 (14)	0.2233 (19)	0.122 (6)	0.411 (6)
H13D	0.2463	0.2068	0.2064	0.183*	0.411 (6)
H13E	0.3288	0.1493	0.2245	0.183*	0.411 (6)
H13F	0.3434	0.2183	0.2863	0.183*	0.411 (6)
C14A	0.6599 (19)	0.3197 (17)	0.086 (3)	0.078 (3)	0.411 (6)
H14C	0.6508	0.3659	0.1114	0.094*	0.411 (6)
H14D	0.6174	0.3159	0.0218	0.094*	0.411 (6)
C15A	0.7649 (17)	0.3117 (14)	0.0793 (17)	0.086 (3)	0.411 (6)
H15C	0.8074	0.3313	0.1373	0.103*	0.411 (6)
H15D	0.7800	0.2622	0.0775	0.103*	0.411 (6)
C16A	0.7871 (17)	0.3454 (12)	−0.0055 (18)	0.096 (4)	0.411 (6)
H16C	0.7799	0.3105	−0.0566	0.116*	0.411 (6)
H16D	0.8559	0.3589	0.0112	0.116*	0.411 (6)
C17A	0.7295 (17)	0.4075 (11)	−0.0469 (17)	0.122 (6)	0.411 (6)
H17D	0.7522	0.4239	−0.1022	0.182*	0.411 (6)
H17E	0.6611	0.3951	−0.0668	0.182*	0.411 (6)
H17F	0.7376	0.4437	0.0014	0.182*	0.411 (6)
C18A	0.6668 (17)	0.1949 (11)	0.1299 (17)	0.085 (3)	0.411 (6)
H18C	0.6748	0.1657	0.1875	0.101*	0.411 (6)
H18D	0.7303	0.1989	0.1131	0.101*	0.411 (6)
C19A	0.5922 (15)	0.1613 (10)	0.0455 (16)	0.093 (4)	0.411 (6)
H19C	0.5261	0.1680	0.0551	0.112*	0.411 (6)
H19D	0.5962	0.1837	−0.0151	0.112*	0.411 (6)
C20A	0.6129 (17)	0.0838 (10)	0.0397 (16)	0.100 (4)	0.411 (6)
H20C	0.5592	0.0566	0.0541	0.119*	0.411 (6)
H20D	0.6730	0.0715	0.0863	0.119*	0.411 (6)
C21A	0.6239 (17)	0.0679 (13)	−0.0686 (16)	0.119 (7)	0.411 (6)
H21D	0.6371	0.0190	−0.0748	0.179*	0.411 (6)
H21E	0.5640	0.0802	−0.1141	0.179*	0.411 (6)
H21F	0.6773	0.0950	−0.0820	0.179*	0.411 (6)
C22A	0.676 (3)	0.2786 (15)	0.2551 (19)	0.082 (3)	0.411 (6)
H22C	0.6483	0.2451	0.2928	0.099*	0.411 (6)
H22D	0.7461	0.2682	0.2658	0.099*	0.411 (6)
C23A	0.666 (4)	0.3485 (15)	0.295 (2)	0.090 (3)	0.411 (6)
H23C	0.5981	0.3576	0.2950	0.108*	0.411 (6)
H23D	0.6884	0.3838	0.2552	0.108*	0.411 (6)
C24A	0.728 (2)	0.3515 (10)	0.3967 (19)	0.100 (4)	0.411 (6)
H24C	0.7068	0.3153	0.4356	0.120*	0.411 (6)
H24D	0.7963	0.3428	0.3961	0.120*	0.411 (6)
C25A	0.7188 (16)	0.4207 (9)	0.4402 (15)	0.102 (5)	0.411 (6)
H25D	0.7586	0.4219	0.5054	0.153*	0.411 (6)
H25E	0.7406	0.4564	0.4020	0.153*	0.411 (6)
H25F	0.6512	0.4289	0.4416	0.153*	0.411 (6)

### Bis(tetra-n-butylammonium) µ-oxalato-bis[bis(1,1,1,4,4,4-hexafluorobut-2-ene-2,3-dithiolato)molybdate(IV)] (k10171_sq) Atomic displacement parameters (Å^2^)

**Table:**

	*U*^11^	*U*^22^	*U*^33^	*U*^12^	*U*^13^	*U*^23^
Mo1	0.0487 (3)	0.0358 (3)	0.0643 (3)	0.0027 (2)	0.0008 (2)	0.0017 (2)
S1	0.0684 (10)	0.0535 (9)	0.0773 (11)	0.0068 (8)	0.0199 (9)	0.0045 (8)
S2	0.0624 (10)	0.0432 (8)	0.0890 (13)	−0.0048 (8)	−0.0006 (9)	0.0076 (8)
S3	0.0642 (10)	0.0487 (9)	0.0731 (11)	0.0066 (8)	0.0075 (9)	−0.0029 (8)
S4	0.0544 (9)	0.0455 (8)	0.0722 (11)	−0.0018 (7)	0.0037 (8)	−0.0027 (8)
F1	0.178 (5)	0.104 (4)	0.122 (4)	0.017 (4)	0.085 (4)	0.010 (3)
F2	0.174 (5)	0.151 (5)	0.109 (4)	0.072 (4)	0.067 (4)	0.053 (4)
F3	0.121 (4)	0.136 (5)	0.198 (6)	0.014 (4)	0.102 (4)	0.039 (4)
F4	0.060 (3)	0.134 (5)	0.384 (11)	−0.021 (3)	−0.014 (4)	0.136 (6)
F5	0.170 (5)	0.069 (3)	0.153 (5)	−0.045 (3)	0.031 (4)	0.017 (3)
F6	0.106 (4)	0.095 (3)	0.167 (5)	0.004 (3)	0.053 (3)	0.058 (4)
F7	0.159 (5)	0.079 (3)	0.270 (8)	−0.004 (3)	0.141 (5)	−0.048 (4)
F8	0.106 (4)	0.177 (6)	0.145 (5)	0.062 (4)	0.060 (4)	0.034 (4)
F9	0.194 (6)	0.100 (3)	0.134 (4)	0.045 (4)	0.106 (4)	0.038 (3)
F10	0.108 (4)	0.167 (6)	0.174 (6)	−0.052 (4)	0.057 (4)	0.002 (4)
F11	0.073 (3)	0.067 (3)	0.208 (6)	−0.018 (2)	−0.005 (3)	−0.008 (3)
F12	0.077 (3)	0.096 (3)	0.187 (5)	−0.022 (3)	−0.033 (3)	0.049 (4)
O1	0.054 (2)	0.038 (2)	0.066 (3)	0.0067 (18)	−0.005 (2)	−0.0040 (19)
O2	0.056 (2)	0.032 (2)	0.073 (3)	0.0048 (19)	−0.007 (2)	−0.0031 (19)
C1	0.061 (4)	0.070 (5)	0.098 (6)	0.011 (4)	0.029 (4)	0.027 (4)
C2	0.055 (4)	0.055 (4)	0.100 (6)	−0.001 (3)	0.012 (4)	0.019 (4)
C3	0.107 (7)	0.088 (6)	0.129 (8)	0.022 (6)	0.064 (6)	0.028 (6)
C4	0.074 (6)	0.068 (5)	0.146 (9)	0.004 (4)	0.009 (5)	0.038 (6)
C5	0.064 (4)	0.054 (4)	0.077 (5)	0.009 (3)	0.022 (4)	0.015 (3)
C6	0.055 (4)	0.050 (3)	0.083 (5)	0.001 (3)	0.010 (3)	0.014 (3)
C7	0.094 (6)	0.063 (5)	0.110 (7)	0.017 (4)	0.049 (5)	0.018 (5)
C8	0.056 (4)	0.069 (5)	0.126 (7)	−0.003 (4)	0.018 (5)	0.013 (5)
C9	0.042 (3)	0.038 (3)	0.072 (4)	−0.001 (3)	0.006 (3)	0.000 (3)
N1	0.056 (3)	0.057 (3)	0.104 (4)	0.003 (3)	−0.003 (3)	0.023 (3)
C10	0.060 (4)	0.066 (5)	0.112 (6)	−0.002 (4)	0.000 (4)	0.023 (5)
C11	0.069 (4)	0.076 (6)	0.125 (7)	−0.010 (5)	0.005 (5)	0.021 (5)
C12	0.075 (5)	0.095 (7)	0.132 (8)	−0.014 (5)	0.006 (6)	0.020 (6)
C13	0.098 (9)	0.139 (10)	0.145 (11)	0.017 (8)	−0.008 (10)	−0.022 (10)
C14	0.062 (4)	0.066 (5)	0.100 (5)	0.007 (4)	0.012 (4)	0.015 (4)
C15	0.073 (4)	0.076 (6)	0.102 (5)	0.011 (4)	0.019 (5)	0.001 (5)
C16	0.077 (5)	0.079 (6)	0.098 (6)	0.008 (5)	0.023 (5)	−0.012 (5)
C17	0.112 (9)	0.117 (9)	0.091 (9)	−0.016 (8)	0.025 (8)	−0.019 (8)
C18	0.066 (5)	0.060 (4)	0.114 (5)	0.006 (4)	−0.011 (4)	0.013 (4)
C19	0.068 (6)	0.068 (5)	0.125 (5)	0.001 (5)	−0.023 (5)	0.005 (5)
C20	0.079 (7)	0.074 (6)	0.131 (6)	−0.001 (6)	−0.025 (6)	−0.004 (5)
C21	0.104 (11)	0.094 (9)	0.147 (10)	−0.005 (9)	−0.021 (9)	−0.012 (8)
C22	0.071 (4)	0.064 (5)	0.102 (5)	0.003 (4)	−0.003 (4)	0.024 (4)
C23	0.086 (5)	0.072 (6)	0.101 (5)	0.008 (5)	0.001 (4)	0.021 (5)
C24	0.103 (6)	0.080 (7)	0.111 (6)	0.012 (6)	−0.003 (5)	0.021 (6)
C25	0.128 (9)	0.098 (9)	0.121 (9)	0.001 (8)	−0.001 (9)	−0.009 (8)
N1A	0.057 (3)	0.059 (4)	0.104 (4)	0.003 (3)	−0.002 (3)	0.021 (3)
C10A	0.059 (4)	0.065 (5)	0.113 (6)	0.000 (4)	0.001 (5)	0.024 (5)
C11A	0.066 (5)	0.076 (6)	0.118 (7)	−0.008 (5)	0.003 (6)	0.023 (5)
C12A	0.069 (6)	0.082 (7)	0.126 (8)	−0.010 (6)	0.005 (7)	0.015 (7)
C13A	0.087 (10)	0.129 (11)	0.144 (12)	0.005 (9)	0.015 (11)	0.026 (11)
C14A	0.063 (4)	0.066 (5)	0.101 (5)	0.006 (4)	0.010 (4)	0.016 (5)
C15A	0.073 (5)	0.078 (6)	0.105 (6)	0.010 (5)	0.019 (5)	0.005 (5)
C16A	0.085 (6)	0.094 (7)	0.110 (7)	0.012 (6)	0.021 (6)	0.005 (6)
C17A	0.122 (11)	0.126 (11)	0.119 (11)	0.002 (10)	0.032 (10)	0.012 (10)
C18A	0.065 (5)	0.058 (5)	0.114 (5)	0.004 (5)	−0.012 (5)	0.014 (5)
C19A	0.072 (7)	0.066 (5)	0.122 (6)	0.000 (5)	−0.019 (6)	0.008 (5)
C20A	0.074 (8)	0.073 (6)	0.128 (7)	−0.001 (6)	−0.025 (7)	0.000 (6)
C21A	0.080 (12)	0.099 (10)	0.153 (11)	0.006 (10)	−0.026 (11)	−0.015 (10)
C22A	0.071 (5)	0.064 (5)	0.102 (5)	0.005 (5)	−0.002 (4)	0.022 (5)
C23A	0.086 (5)	0.071 (6)	0.103 (5)	0.007 (6)	0.000 (5)	0.022 (5)
C24A	0.101 (6)	0.077 (7)	0.111 (6)	0.009 (6)	−0.002 (6)	0.020 (7)
C25A	0.127 (11)	0.069 (9)	0.101 (10)	−0.001 (9)	0.008 (9)	0.002 (9)

### Bis(tetra-n-butylammonium) µ-oxalato-bis[bis(1,1,1,4,4,4-hexafluorobut-2-ene-2,3-dithiolato)molybdate(IV)] (k10171_sq) Geometric parameters (Å, º)

**Table:**

Mo1—O1	2.165 (4)	C20—H20B	0.9900
Mo1—O2^i^	2.168 (4)	C21—H21A	0.9800
Mo1—S3	2.3116 (18)	C21—H21B	0.9800
Mo1—S1	2.3148 (19)	C21—H21C	0.9800
Mo1—S4	2.3186 (16)	C22—C23	1.503 (13)
Mo1—S2	2.3216 (16)	C22—H22A	0.9900
S1—C1	1.749 (7)	C22—H22B	0.9900
S2—C2	1.742 (8)	C23—C24	1.520 (14)
S3—C5	1.724 (7)	C23—H23A	0.9900
S4—C6	1.734 (7)	C23—H23B	0.9900
F1—C3	1.333 (10)	C24—C25	1.51 (2)
F2—C3	1.319 (10)	C24—H24A	0.9900
F3—C3	1.312 (10)	C24—H24B	0.9900
F4—C4	1.283 (9)	C25—H25A	0.9800
F5—C4	1.325 (10)	C25—H25B	0.9800
F6—C4	1.318 (10)	C25—H25C	0.9800
F7—C7	1.288 (9)	N1A—C14A	1.512 (14)
F8—C7	1.335 (9)	N1A—C10A	1.519 (14)
F9—C7	1.277 (8)	N1A—C22A	1.519 (14)
F10—C8	1.333 (9)	N1A—C18A	1.530 (14)
F11—C8	1.320 (9)	C10A—C11A	1.507 (16)
F12—C8	1.290 (9)	C10A—H10C	0.9900
O1—C9	1.254 (6)	C10A—H10D	0.9900
O2—C9	1.255 (6)	C11A—C12A	1.538 (16)
O2—Mo1^i^	2.168 (4)	C11A—H11C	0.9900
C1—C2	1.318 (10)	C11A—H11D	0.9900
C1—C3	1.537 (11)	C12A—C13A	1.510 (19)
C2—C4	1.507 (10)	C12A—H12C	0.9900
C5—C6	1.357 (9)	C12A—H12D	0.9900
C5—C7	1.517 (10)	C13A—H13D	0.9800
C6—C8	1.508 (9)	C13A—H13E	0.9800
C9—C9^i^	1.509 (11)	C13A—H13F	0.9800
N1—C14	1.509 (11)	C14A—C15A	1.529 (16)
N1—C18	1.522 (12)	C14A—H14C	0.9900
N1—C10	1.532 (11)	C14A—H14D	0.9900
N1—C22	1.539 (12)	C15A—C16A	1.483 (17)
C10—C11	1.519 (13)	C15A—H15C	0.9900
C10—H10A	0.9900	C15A—H15D	0.9900
C10—H10B	0.9900	C16A—C17A	1.506 (18)
C11—C12	1.547 (14)	C16A—H16C	0.9900
C11—H11A	0.9900	C16A—H16D	0.9900
C11—H11B	0.9900	C17A—H17D	0.9800
C12—C13	1.476 (16)	C17A—H17E	0.9800
C12—H12A	0.9900	C17A—H17F	0.9800
C12—H12B	0.9900	C18A—C19A	1.564 (17)
C13—H13A	0.9800	C18A—H18C	0.9900
C13—H13B	0.9800	C18A—H18D	0.9900
C13—H13C	0.9800	C19A—C20A	1.544 (16)
C14—C15	1.538 (14)	C19A—H19C	0.9900
C14—H14A	0.9900	C19A—H19D	0.9900
C14—H14B	0.9900	C20A—C21A	1.633 (19)
C15—C16	1.518 (14)	C20A—H20C	0.9900
C15—H15A	0.9900	C20A—H20D	0.9900
C15—H15B	0.9900	C21A—H21D	0.9800
C16—C17	1.564 (15)	C21A—H21E	0.9800
C16—H16A	0.9900	C21A—H21F	0.9800
C16—H16B	0.9900	C22A—C23A	1.498 (16)
C17—H17A	0.9800	C22A—H22C	0.9900
C17—H17B	0.9800	C22A—H22D	0.9900
C17—H17C	0.9800	C23A—C24A	1.527 (17)
C18—C19	1.574 (15)	C23A—H23C	0.9900
C18—H18A	0.9900	C23A—H23D	0.9900
C18—H18B	0.9900	C24A—C25A	1.51 (2)
C19—C20	1.548 (13)	C24A—H24C	0.9900
C19—H19A	0.9900	C24A—H24D	0.9900
C19—H19B	0.9900	C25A—H25D	0.9800
C20—C21	1.608 (15)	C25A—H25E	0.9800
C20—H20A	0.9900	C25A—H25F	0.9800
			
O1—Mo1—O2^i^	74.40 (14)	C20—C21—H21A	109.5
O1—Mo1—S3	132.28 (12)	C20—C21—H21B	109.5
O2^i^—Mo1—S3	83.24 (11)	H21A—C21—H21B	109.5
O1—Mo1—S1	84.60 (12)	C20—C21—H21C	109.5
O2^i^—Mo1—S1	133.48 (12)	H21A—C21—H21C	109.5
S3—Mo1—S1	137.72 (6)	H21B—C21—H21C	109.5
O1—Mo1—S4	83.76 (11)	C23—C22—N1	116.1 (11)
O2^i^—Mo1—S4	133.74 (12)	C23—C22—H22A	108.3
S3—Mo1—S4	82.25 (6)	N1—C22—H22A	108.3
S1—Mo1—S4	82.60 (6)	C23—C22—H22B	108.3
O1—Mo1—S2	133.64 (11)	N1—C22—H22B	108.3
O2^i^—Mo1—S2	83.25 (11)	H22A—C22—H22B	107.4
S3—Mo1—S2	82.88 (6)	C22—C23—C24	115.3 (12)
S1—Mo1—S2	82.21 (7)	C22—C23—H23A	108.4
S4—Mo1—S2	137.48 (6)	C24—C23—H23A	108.4
C1—S1—Mo1	108.8 (3)	C22—C23—H23B	108.4
C2—S2—Mo1	109.4 (2)	C24—C23—H23B	108.4
C5—S3—Mo1	109.9 (2)	H23A—C23—H23B	107.5
C6—S4—Mo1	109.2 (2)	C25—C24—C23	111.7 (19)
C9—O1—Mo1	116.0 (3)	C25—C24—H24A	109.3
C9—O2—Mo1^i^	117.0 (3)	C23—C24—H24A	109.3
C2—C1—C3	125.5 (7)	C25—C24—H24B	109.3
C2—C1—S1	120.3 (6)	C23—C24—H24B	109.3
C3—C1—S1	114.2 (6)	H24A—C24—H24B	107.9
C1—C2—C4	126.2 (8)	C24—C25—H25A	109.5
C1—C2—S2	119.1 (5)	C24—C25—H25B	109.5
C4—C2—S2	114.6 (7)	H25A—C25—H25B	109.5
F3—C3—F2	107.7 (8)	C24—C25—H25C	109.5
F3—C3—F1	106.8 (7)	H25A—C25—H25C	109.5
F2—C3—F1	106.9 (9)	H25B—C25—H25C	109.5
F3—C3—C1	112.3 (9)	C14A—N1A—C10A	103.8 (13)
F2—C3—C1	111.8 (7)	C14A—N1A—C22A	112.4 (16)
F1—C3—C1	111.0 (7)	C10A—N1A—C22A	112.7 (16)
F4—C4—F6	108.0 (8)	C14A—N1A—C18A	111.1 (15)
F4—C4—F5	108.8 (9)	C10A—N1A—C18A	112.3 (14)
F6—C4—F5	102.0 (7)	C22A—N1A—C18A	104.6 (14)
F4—C4—C2	112.7 (7)	C11A—C10A—N1A	115.9 (14)
F6—C4—C2	113.6 (8)	C11A—C10A—H10C	108.3
F5—C4—C2	111.2 (8)	N1A—C10A—H10C	108.3
C6—C5—C7	126.5 (6)	C11A—C10A—H10D	108.3
C6—C5—S3	119.1 (5)	N1A—C10A—H10D	108.3
C7—C5—S3	114.5 (5)	H10C—C10A—H10D	107.4
C5—C6—C8	126.4 (7)	C10A—C11A—C12A	109.1 (13)
C5—C6—S4	119.4 (5)	C10A—C11A—H11C	109.9
C8—C6—S4	114.2 (5)	C12A—C11A—H11C	109.9
F9—C7—F7	110.1 (8)	C10A—C11A—H11D	109.9
F9—C7—F8	105.1 (7)	C12A—C11A—H11D	109.9
F7—C7—F8	103.7 (7)	H11C—C11A—H11D	108.3
F9—C7—C5	113.1 (6)	C13A—C12A—C11A	108.5 (15)
F7—C7—C5	111.9 (7)	C13A—C12A—H12C	110.0
F8—C7—C5	112.3 (7)	C11A—C12A—H12C	110.0
F12—C8—F11	106.8 (8)	C13A—C12A—H12D	110.0
F12—C8—F10	107.5 (7)	C11A—C12A—H12D	110.0
F11—C8—F10	102.8 (7)	H12C—C12A—H12D	108.4
F12—C8—C6	113.4 (6)	C12A—C13A—H13D	109.5
F11—C8—C6	113.3 (6)	C12A—C13A—H13E	109.5
F10—C8—C6	112.4 (7)	H13D—C13A—H13E	109.5
O1—C9—O2	127.5 (5)	C12A—C13A—H13F	109.5
O1—C9—C9^i^	117.3 (6)	H13D—C13A—H13F	109.5
O2—C9—C9^i^	115.3 (6)	H13E—C13A—H13F	109.5
C14—N1—C18	113.4 (12)	N1A—C14A—C15A	112.1 (16)
C14—N1—C10	107.7 (10)	N1A—C14A—H14C	109.2
C18—N1—C10	109.6 (10)	C15A—C14A—H14C	109.2
C14—N1—C22	110.5 (11)	N1A—C14A—H14D	109.2
C18—N1—C22	107.6 (10)	C15A—C14A—H14D	109.2
C10—N1—C22	107.9 (11)	H14C—C14A—H14D	107.9
C11—C10—N1	116.8 (11)	C16A—C15A—C14A	113.8 (16)
C11—C10—H10A	108.1	C16A—C15A—H15C	108.8
N1—C10—H10A	108.1	C14A—C15A—H15C	108.8
C11—C10—H10B	108.1	C16A—C15A—H15D	108.8
N1—C10—H10B	108.1	C14A—C15A—H15D	108.8
H10A—C10—H10B	107.3	H15C—C15A—H15D	107.7
C10—C11—C12	111.2 (11)	C15A—C16A—C17A	118.9 (18)
C10—C11—H11A	109.4	C15A—C16A—H16C	107.6
C12—C11—H11A	109.4	C17A—C16A—H16C	107.6
C10—C11—H11B	109.4	C15A—C16A—H16D	107.6
C12—C11—H11B	109.4	C17A—C16A—H16D	107.6
H11A—C11—H11B	108.0	H16C—C16A—H16D	107.0
C13—C12—C11	109.5 (13)	C16A—C17A—H17D	109.5
C13—C12—H12A	109.8	C16A—C17A—H17E	109.5
C11—C12—H12A	109.8	H17D—C17A—H17E	109.5
C13—C12—H12B	109.8	C16A—C17A—H17F	109.5
C11—C12—H12B	109.8	H17D—C17A—H17F	109.5
H12A—C12—H12B	108.2	H17E—C17A—H17F	109.5
C12—C13—H13A	109.5	N1A—C18A—C19A	109.4 (14)
C12—C13—H13B	109.5	N1A—C18A—H18C	109.8
H13A—C13—H13B	109.5	C19A—C18A—H18C	109.8
C12—C13—H13C	109.5	N1A—C18A—H18D	109.8
H13A—C13—H13C	109.5	C19A—C18A—H18D	109.8
H13B—C13—H13C	109.5	H18C—C18A—H18D	108.2
N1—C14—C15	116.8 (13)	C20A—C19A—C18A	110.4 (14)
N1—C14—H14A	108.1	C20A—C19A—H19C	109.6
C15—C14—H14A	108.1	C18A—C19A—H19C	109.6
N1—C14—H14B	108.1	C20A—C19A—H19D	109.6
C15—C14—H14B	108.1	C18A—C19A—H19D	109.6
H14A—C14—H14B	107.3	H19C—C19A—H19D	108.1
C16—C15—C14	112.3 (11)	C19A—C20A—C21A	107.4 (16)
C16—C15—H15A	109.1	C19A—C20A—H20C	110.2
C14—C15—H15A	109.1	C21A—C20A—H20C	110.2
C16—C15—H15B	109.1	C19A—C20A—H20D	110.2
C14—C15—H15B	109.1	C21A—C20A—H20D	110.2
H15A—C15—H15B	107.9	H20C—C20A—H20D	108.5
C15—C16—C17	108.5 (12)	C20A—C21A—H21D	109.5
C15—C16—H16A	110.0	C20A—C21A—H21E	109.5
C17—C16—H16A	110.0	H21D—C21A—H21E	109.5
C15—C16—H16B	110.0	C20A—C21A—H21F	109.5
C17—C16—H16B	110.0	H21D—C21A—H21F	109.5
H16A—C16—H16B	108.4	H21E—C21A—H21F	109.5
C16—C17—H17A	109.5	C23A—C22A—N1A	118.1 (16)
C16—C17—H17B	109.5	C23A—C22A—H22C	107.8
H17A—C17—H17B	109.5	N1A—C22A—H22C	107.8
C16—C17—H17C	109.5	C23A—C22A—H22D	107.8
H17A—C17—H17C	109.5	N1A—C22A—H22D	107.8
H17B—C17—H17C	109.5	H22C—C22A—H22D	107.1
N1—C18—C19	113.4 (11)	C22A—C23A—C24A	108.6 (16)
N1—C18—H18A	108.9	C22A—C23A—H23C	110.0
C19—C18—H18A	108.9	C24A—C23A—H23C	110.0
N1—C18—H18B	108.9	C22A—C23A—H23D	110.0
C19—C18—H18B	108.9	C24A—C23A—H23D	110.0
H18A—C18—H18B	107.7	H23C—C23A—H23D	108.4
C20—C19—C18	109.6 (11)	C25A—C24A—C23A	110.4 (17)
C20—C19—H19A	109.7	C25A—C24A—H24C	109.6
C18—C19—H19A	109.7	C23A—C24A—H24C	109.6
C20—C19—H19B	109.7	C25A—C24A—H24D	109.6
C18—C19—H19B	109.7	C23A—C24A—H24D	109.6
H19A—C19—H19B	108.2	H24C—C24A—H24D	108.1
C19—C20—C21	110.4 (11)	C24A—C25A—H25D	109.5
C19—C20—H20A	109.6	C24A—C25A—H25E	109.5
C21—C20—H20A	109.6	H25D—C25A—H25E	109.5
C19—C20—H20B	109.6	C24A—C25A—H25F	109.5
C21—C20—H20B	109.6	H25D—C25A—H25F	109.5
H20A—C20—H20B	108.1	H25E—C25A—H25F	109.5
			
Mo1—S1—C1—C2	−3.8 (6)	Mo1^i^—O2—C9—O1	179.0 (5)
Mo1—S1—C1—C3	179.4 (5)	Mo1^i^—O2—C9—C9^i^	−1.7 (8)
C3—C1—C2—C4	−4.0 (12)	C14—N1—C10—C11	−172.6 (18)
S1—C1—C2—C4	179.7 (6)	C18—N1—C10—C11	63.6 (18)
C3—C1—C2—S2	177.6 (6)	C22—N1—C10—C11	−53.3 (18)
S1—C1—C2—S2	1.3 (8)	N1—C10—C11—C12	−172.9 (13)
Mo1—S2—C2—C1	1.9 (6)	C10—C11—C12—C13	89 (2)
Mo1—S2—C2—C4	−176.6 (5)	C18—N1—C14—C15	−74 (3)
C2—C1—C3—F3	45.3 (11)	C10—N1—C14—C15	165 (2)
S1—C1—C3—F3	−138.2 (6)	C22—N1—C14—C15	47 (3)
C2—C1—C3—F2	−76.0 (11)	N1—C14—C15—C16	−177.5 (16)
S1—C1—C3—F2	100.6 (8)	C14—C15—C16—C17	−65 (2)
C2—C1—C3—F1	164.8 (8)	C14—N1—C18—C19	−38.5 (19)
S1—C1—C3—F1	−18.6 (10)	C10—N1—C18—C19	81.9 (17)
C1—C2—C4—F4	−84.2 (12)	C22—N1—C18—C19	−161.0 (17)
S2—C2—C4—F4	94.3 (10)	N1—C18—C19—C20	176.0 (12)
C1—C2—C4—F6	39.0 (11)	C18—C19—C20—C21	137.9 (18)
S2—C2—C4—F6	−142.6 (7)	C14—N1—C22—C23	61 (3)
C1—C2—C4—F5	153.4 (8)	C18—N1—C22—C23	−174 (2)
S2—C2—C4—F5	−28.2 (9)	C10—N1—C22—C23	−56 (3)
Mo1—S3—C5—C6	−3.1 (6)	N1—C22—C23—C24	175 (2)
Mo1—S3—C5—C7	176.5 (4)	C22—C23—C24—C25	68 (3)
C7—C5—C6—C8	2.0 (11)	C14A—N1A—C10A—C11A	177 (3)
S3—C5—C6—C8	−178.4 (6)	C22A—N1A—C10A—C11A	−61 (3)
C7—C5—C6—S4	−179.3 (5)	C18A—N1A—C10A—C11A	57 (3)
S3—C5—C6—S4	0.3 (8)	N1A—C10A—C11A—C12A	178 (2)
Mo1—S4—C6—C5	2.7 (6)	C10A—C11A—C12A—C13A	−161 (2)
Mo1—S4—C6—C8	−178.5 (5)	C10A—N1A—C14A—C15A	−166 (3)
C6—C5—C7—F9	77.8 (10)	C22A—N1A—C14A—C15A	72 (4)
S3—C5—C7—F9	−101.8 (7)	C18A—N1A—C14A—C15A	−45 (3)
C6—C5—C7—F7	−157.1 (8)	N1A—C14A—C15A—C16A	160 (2)
S3—C5—C7—F7	23.3 (9)	C14A—C15A—C16A—C17A	29 (4)
C6—C5—C7—F8	−40.9 (10)	C14A—N1A—C18A—C19A	−85 (3)
S3—C5—C7—F8	139.5 (6)	C10A—N1A—C18A—C19A	31 (3)
C5—C6—C8—F12	83.2 (10)	C22A—N1A—C18A—C19A	153 (3)
S4—C6—C8—F12	−95.5 (7)	N1A—C18A—C19A—C20A	−165.9 (18)
C5—C6—C8—F11	−154.9 (7)	C18A—C19A—C20A—C21A	−126 (2)
S4—C6—C8—F11	26.4 (9)	C14A—N1A—C22A—C23A	51 (4)
C5—C6—C8—F10	−38.9 (10)	C10A—N1A—C22A—C23A	−66 (4)
S4—C6—C8—F10	142.4 (6)	C18A—N1A—C22A—C23A	172 (4)
Mo1—O1—C9—O2	178.5 (5)	N1A—C22A—C23A—C24A	−173 (3)
Mo1—O1—C9—C9^i^	−0.7 (8)	C22A—C23A—C24A—C25A	−179 (4)

Symmetry code: (i) −*x*+2, −*y*, −*z*+1.
